# Biomechanical analysis of hip, knee, and ankle joint contact forces during squats in elite powerlifters

**DOI:** 10.1371/journal.pone.0327973

**Published:** 2025-07-24

**Authors:** Alexander Pürzel, Paul Kaufmann, Willi Koller, Elias Kaj Wallnöfer, Arnold Baca, Hans Kainz

**Affiliations:** 1 Neuromechanics Research Group, University of Vienna, Vienna, Austria; 2 Vienna Doctoral School of Pharmaceutical, Nutritional and Sport Sciences, University of Vienna, Vienna, Austria; 3 Centre for Sport Science and University Sports, University of Vienna, Vienna, Austria; 4 Université Côte d’Azur, LAMHESS, Nice, France; University of Memphis, UNITED STATES OF AMERICA

## Abstract

The squat is one of three lifts within the sport of powerlifting. This study examined how increasing intensity in the squat affects joint contact forces in elite powerlifters. Twenty-nine Austrian top-ranked powerlifters (16 male, 13 female) performed squats at 70% to 90% of their one-repetition maximum (1-RM). 3D motion capture and force plate data were used to estimate joint contact forces using musculoskeletal modelling. In contrast to the hip and ankle joints, which exhibited peak resultant joint contact forces in the deepest squat positions, the tibiofemoral and patellofemoral joints maintained consistently high loads over a broad portion of the squat cycle. During large parts of the concentric phase, the resultant joint contact forces did not significantly differ between intensity conditions, with the exception of the hip joint contact force. At 90% 1-RM, average peak joint contact forces reached 15.5 ± 3.0 times body weight (BW), 23.2 ± 3.9 BW, 26.7 ± 4.3 BW, and 11.5 ± 2.2 BW for the hip, tibiofemoral, patellofemoral, and ankle joint, respectively. The high and sustained joint contact forces observed in our study emphasise the need for load management strategies to optimise performance and reduce injury risk. These insights offer a valuable foundation for tailoring strength training programs and supporting long-term joint health in high-performance athletes.

## Introduction

In the sport of powerlifting, the squat represents the initial of three exercises, followed by the bench press and the deadlift. The objective is to achieve the highest possible weight in each of the lifts. Biomechanical and training science studies offer practical relevant insights in the squat for coaches and athletes alike. In addition to the advancement of knowledge concerning muscle forces [1–3], optimal technique [4–7] and training planning [8,9], it is imperative to recognise that squats can exert considerable forces on the involved joints – without [10–12] and with [13–15] additional external load.

In recent years, there has been a notable increase in the number of musculoskeletal modelling studies analysing joint contact forces during squats [16,17]. Simulations of bodyweight squats estimated tibiofemoral joint contact forces of 2.5 ± 0.3 body weight (BW) [17] and hip joint contact forces ranging from 2.5 to 3.0 BW [18]. In contrast, single-leg squats produced significantly higher hip joint contact forces, reaching 8.4 ± 0.4 BW [16]. Squats performed with an additional weight of 53 ± 12 kg resulted in patellofemoral joint contact forces between 6.0 BW and 7.1 BW [15].

Despite these recent insights, joint contact forces are often overlooked in the development and assessment of training programmes. However, if joint contact forces during exercises are beyond the physiological limits of joint structures, the training might exacerbate existing joint pathologies or even result in serious injuries. While the exact magnitude of force that is detrimental to joint structures, including ligaments, menisci and articular cartilage, remains uncertain [13,19], excessive stress on these structures can result in degenerative changes [20,21]. Conversely, appropriate compressive forces in the tibiofemoral joint may exert a hypertrophic effect on the cartilage [22]. The precise level of the joint contact forces and other load variables necessary for this effect remain to be determined through further research. Furthermore, compressive forces are of paramount importance for stabilising the knee joint, as they counteract anterior-posterior shear forces and, therefore, reduce the anterior translation of the tibia in relation to the femur while also contributing to frontal plane stability by limiting varus and valgus motion [23].

Worth noting, no study quantified joint contact forces during squats in powerlifting – particularly in a cohort of elite powerlifters with external barbell loads beyond 2 times BW. In order to enhance injury prevention, performance as well as rehabilitation, it is imperative for coaches and athletes to know how high joint contact forces are during squats. A deeper understanding of the magnitude and timing of joint contact forces during squats is essential for informed training planning, as these forces directly influence the mechanical loading of passive joint structures such as cartilage – thereby influencing their long-term adaptation, integrity, or degeneration.

The aim of this study was to examine the influence of an increasing intensity from 70% to 90% of the one-repetition maximum (1-RM) in the squat on joint contact forces of the hip, knee, and ankle joints of elite powerlifters. Two hypotheses were subsequently proposed: (a) the peak joint contact forces in each anatomical direction (anterior-posterior, medio-lateral, vertical, resultant) of each joint (hip, tibiofemoral, patellofemoral, ankle) exhibit a significant increase with increasing intensity; and (b) the joint contact forces exhibit a significant increase during the whole squat movement (expressed as % of the squat cycle) of the analysed joints with increasing intensity.

## Materials and methods

### Participants

The study comprised a cohort of 29 healthy elite powerlifters (13 female, 16 male) spanning various weight classes, as detailed in Table 1. The participants were either current members of the Austrian national powerlifting team, competing internationally in Western European, European, and/or World Championships (n = 14), or had achieved a top three ranking at an Austrian powerlifting championship between 2019 and 2022 (n = 15). Due to the focus on elite powerlifters, who represent a narrowly defined and difficult to access population, the sample size of 29 participants is consistent with or even exceeds that of previous musculoskeletal modelling studies in strength-trained individuals [1,3]. All participants were required to maintain an active membership of the Austrian Powerlifting Federation, which mandates a medical examination and were recruited between 1 July and 30 September 2022. They had no current or previous musculoskeletal injuries that would hinder their ability to perform the squat. The study was approved by the Research Ethics Committee of the authors’ institution (Ethics Committee of the University of Vienna, Ref. No.: 00771). Prior to their participation in the research study, the participants were provided with comprehensive information regarding the procedures, potential benefits, and any associated risks. Subsequent to an explanation of the research details, each participant provided written informed consent, indicating a clear understanding of the information presented. Furthermore, they were instructed to refrain from engaging in strenuous lower-body exercises for a minimum of three days prior to the data collection.

**Table 1:** Participant descriptive statistics. Wilks score and IPF-GL points (International Powerlifting Federation Good Lift points), as indicated in the table, are both metrics that quantify the relationship between body weight and performance. A higher value indicates a higher relative performance.

Descriptive	Mean	SD
**Body mass (kg)**	83.1	19.4
**Height (cm)**	171.0	10.1
**Age (years)**	26.1	5.4
**Years of powerlifting training**	8.4	4.1
**Relative squat performance (xBM)**	2.4	0.4
**Wilks score**	418.7	40.0
**IPF-GL points**	86.3	8.5

IPF-GL points = International Powerlifting Federation Good Lift points. BM = body mass. SD = standard deviation.

### Three-dimensional motion capturing

**Fig 1:**
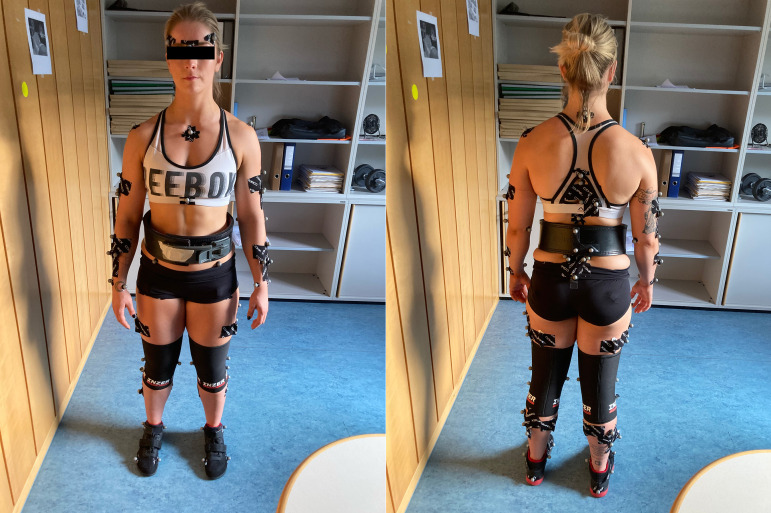
Marker placement on the athletes. temporal right/left (ri/le), glabella, acromion ri/le, manubrium, proc. xiphoideus, epicondylus lat. ri/le, epicondylus med. ri/le, ulna distalis ri/le, radius distalis ri/le, upper arm cluster ri/le, lower arm cluster ri/le, spina iliaca anterior superior (SIAS) ri/le, spina iliaca posterior superior (SIPS) ri/le, condylus med. ri/le, condylus lat. ri/le, caput fib. ri/le, malleolus lat. ri/le, malleolus med. ri/le, calcaneus ri/le, metatarsus I ri/le, metatarsus V ri/le, thigh cluster ri/le, shank cluster ri/le, cervical vertebra (C)7, thoracic vertebra (T)6, T12, upper back cluster, sacrum cluster, belt cluster, bar ri/le. 20 markers were removed after the static calibration trial. These markers include the epicondylus lat. ri/le, epicondylus med. ri/le, radius distalis ri/le, SIAS ri/le, SIPS ri/le, condylus med. ri/le, condylus lat. ri/le, caput fib. ri/le, malleolus lat. ri/le, and malleolus med. ri/le markers.

A three-dimensional motion capture system comprising 12 cameras (Vicon Motion System, Oxford, UK) was used to capture the trajectories of 73 (static position) and 53 (squat movement) markers on the lower limbs, upper body, and barbell with a recording frequency of 200 Hz (Fig 1). The captured marker data was labelled and processed with a Butterworth fourth-order low-pass filter with a cutoff frequency of 6 Hz using Nexus 2.14.1 software (Vicon Motion System, Oxford, UK). The ground reaction forces were recorded simultaneously at a frequency of 1000 Hz via two separate force plates (Kistler Instrumente, Winterthur, Switzerland). During the squat, participants were permitted to utilise their own equipment, including footwear (with or without elevated heels), knee sleeves, powerlifting belts, and wrist wraps, provided that all equipment complied with the standards of the International Powerlifting Federation (IPF) [24]. The warm-up protocol, nutritional intake prior to and during the testing, and rest intervals between attempts were not subject to any restrictions, thereby enabling the participants to adequately prepare and perform in accordance with their accustomed routines. Each participant performed a single squat at 70%, 75%, 80%, 85%, and 90% of their 1-RM, in accordance with the technique standards of the IPF [25]. It was imperative that the hip joint descended below the knee joint at the lowest point of the squat. To guarantee the safety of the participants, three spotters were present for each squat, and verbal encouragement was provided throughout each attempt.

The 1-RM values were approximated by analysing the participants’ recent training and competition results, incorporating feedback from both the participants and their coaches. This approach was adopted in response to concerns raised by some participants and coaches that performing 1-RM tests might have a detrimental impact on their training and upcoming competition performance.

### Magnetic resonance imaging

In six of the 29 participants, magnetic resonance imaging (MRI) was conducted on the lower limbs using a 3 Tesla MRI scanner (MAGNETOM Vida, Siemens, Berlin/Munich, Germany) with a T1 vibe sequence, providing a voxel resolution of 0.98x0.98x1.0 mm, MRI images were used for assessing muscle volume. The MRI-based muscle volume data from these six participants was used to calculate a mean scaling factor for maximum isometric muscle force, which was then applied to all participants in our study (for details, see section “Calculation of joint contact forces”). Muscle segmentation was performed using the open-source software 3D Slicer (version 5.2.2) [26]. Manual annotations were created with at least ten slices per muscle marked in each anatomical plane to provide a robust basis for interpolation. The remaining layers were interpolated, generating continuous 3D segmentations of the target muscles (mm. vasti, m. rectus femoris, m. semimembranosus, m. gluteus maximus). If interpolation errors were detected, additional slices were manually segmented until an anatomically accurate muscle representation was achieved and final muscle volumes were calculated. For a better illustration of the process, please refer to S5 Fig in the Supporting Information.

### Calculation of joint contact forces

Musculoskeletal modelling in OpenSim 4.4 [27] was used to estimate joint contact forces. All calculations were based on the “Catelli”-model [28], a validated model for high hip and knee flexion. The initial stage of the process involved scaling the base model to the participants’ anthropometry based on the location of the surface markers [29]. In order to ensure that the model is capable of producing necessary internal joint moments to resist the external moments, the maximum isometric muscle forces (F) for all models were adjusted based on their body mass (M) using Equation 1 [30,31].


$$F_{iso,scaled}=F_{iso,generic}*{\left(\frac{M_{scaled}}{M_{generic}}\right)}^{\frac{2}{3}}$$ (1)

Despite this muscle force scaling process, the simulations resulted in reserve actuator forces that were beyond 10% of the net joint moments, thereby producing simulations that were not realistic [32]. In other words, the models were too weak to perform the high load exercises of the elite-level athletes. In order to obtain a more precise estimation of the isometric maximum force of the participants and to make the necessary adjustments to the models, MRI scans of the lower limbs were analysed from six participants. The mm. vasti, m. rectus femoris, m. semimembranosus, and m. gluteus maximus muscles were segmented and their volumes were calculated using the 3D Slicer 5.2.2 [26]. The maximum isometric muscle force was subsequently calculated using Equation 2 [33,34], where $F_{iso}$ represents the muscle’s isometric maximum force, ss means subject specific, V is the muscle volume determined by the MRI scans and $l_{o}^{m}$ is the optimal fibre length (OpenSim 4.4 default [27]). The maximum fibre tension (σ) was set to 105 N cm ⁻ ², a value which falls within the range previously reported in the literature [35]. This choice was based on anatomical cross-sectional areas measured in bodybuilders [36] and was adjusted for muscle pennation angle [36–38] to better reflect the physiological cross-sectional area.


$$F_{iso,ss}=\frac{V_{ss}}{l_{o,ss}^{m}}*\sigma$$ (2)

The maximum isometric force calculated using Equation 1 was found to be three times lower (S obtained with Equation 3) than the corresponding estimate using Equation 2, which was based on MRI-derived muscle volume data.


$$S\approx mean\;\frac{F_{iso,ss}}{F_{iso,scaled}}$$ (3)

Consequently, the final equation (Equation 4) employed for the scaling of isometric forces across all 29 models involved multiplying the initial equation (Equation 1) by factor S (Equation 3), to ensure that the models are capable of withstanding the external loads.


$$F_{iso,scaled\_final}=F_{iso,generic}*{\left(\frac{M_{scaled}}{M_{generic}}\right)}^{\frac{2}{3}}*S$$ (4)

Although Equation 2 was applied only to the muscle volume values of the MRI subgroup (6 athletes), its output was used to define the scaling factor in Equation 4. Given the homogeneity of the participants with respect to their performance level, Equation 4 with S=3 was employed to scale the isometric maximum force for the models of all 29 participants.

After scaling the models to the anthropometry of each participant [29], inverse kinematics and inverse dynamics was employed to calculate joint angles and joint moments, respectively. Subsequently, Static Optimization was employed to estimate muscle forces and activations by minimising the sum of squared muscle activations [27]. Ultimately, Joint Reaction Analysis was conducted to determine the hip, tibiofemoral, patellofemoral, and ankle joint contact forces, considering contributions from muscle forces [39,40], as well as the external loads. Hip joint contact forces and orientations are reported in relation to the local coordinate system of the femur. Tibiofemoral contact forces and orientations are reported in relation to the local coordinate system of the tibia. Patellofemoral contact forces and orientations are expressed in relation to the local coordinate system of the patella. Ankle joint contact forces and orientations are reported in relation to the local coordinate system of the talus.

### Model validation

To validate our simulation results, a qualitative visual comparison of the hip and knee joint contact forces calculated during unloaded squats of one athlete was made with those found on OrthoLoad [41], a public database of hip and knee joint contact forces measured in vivo with instrumented hip and knee implants. Furthermore, for the same participant, electromyography (EMG) data were available and used as an additional validation step for evaluating the plausibility of the simulated neuromuscular control patterns during squatting. For the 70% 1-RM condition, we qualitatively compared EMG signals of m. biceps femoris, m. gluteus maximus, m. rectus femoris, and m. vastus lateralis with muscle activation output from our musculoskeletal simulations (i.e., obtained from Static Optimization).

### Data analyses

The squat cycle in this study was defined as follows: The cycle began when the C7 marker (placed on the seventh cervical vertebra) descended by 2% of its initial vertical distance to the floor, marking 0% of the squat cycle. The lowest point of the squat (50% of the cycle) corresponded to the minimum vertical position of the sacrum marker. The cycle was considered complete (100%) when the C7 marker returned to 98% of its initial vertical height. Joint contact force waveforms were normalized over the squat cycle to 101 data points. The eccentric (0% – 50%) and concentric phases (51% to 100%) were analysed separately and averaged across both legs. Peak joint contact forces were identified for each phase individually.

To detect changes in joint contact force waveforms throughout the squat cycle across different intensity conditions (70%, 75%, 80%, 85%, 90% of 1-RM), Statistical Parametric Mapping (SPM) [42] was used, utilising the SPM1D package [43] in Matlab R2022a (Mathworks Inc., Natick, MA, USA). In instances where a statistically significant result was obtained by the repeated measures ANOVA (*p* ≤ 0.05), a Bonferroni correction was applied for post-hoc pairwise comparisons.

A two-way repeated measures ANOVA was conducted to evaluate the effects of phases (eccentric and concentric) and intensities (70% to 90% of 1-RM) on peak joint contact forces for each joint and anatomical direction. Subsequently, a Bonferroni correction was applied for the purposes of conducting post-hoc pairwise comparisons. This statistical analysis was conducted using JASP (version 0.18.1.0), with a significance level of *p* ≤ 0.05. A post-hoc power analysis (ANOVA: repeated measures, within factors) was conducted using G*Power (version 3.1.9.7) [44], yielding a result of 93.5%.

## Results

### Validation of simulation results

The shape of the hip and knee joint contact force waveforms from our simulations showed a reasonable agreement with the values from instrumented implants (S1 Fig in the Supporting Information) but with higher force magnitudes. Moreover, hip joint contact forces showed similar shape compared to previous reported values from a musculoskeletal modelling study [16] (S2 Fig in the Supporting Information). Estimated muscle activations from Static Optimization showed reasonable agreement with experimental EMG signals for all analysed muscles, except the m. rectus femoris (S3 Fig in the Supporting Information).

### Peak joint contact forces

**Fig 2:**
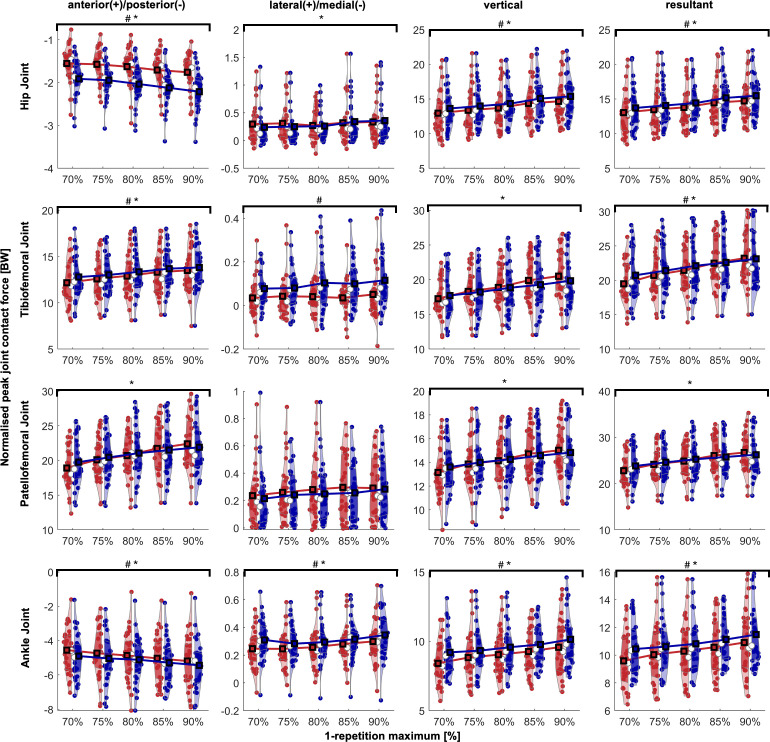
Peak joint contact forces during squats with different intensities. Each coloured circle represents one participant; black squares represent mean values; white circles indicate median values. Shaded areas represent violin plots, illustrating the distribution of data; darker areas indicate quartiles, while lighter areas show the remainder of the distribution; red is eccentric, blue is concentric; # indicates significant difference for phase (eccentric, concentric); * indicates significant difference for intensity.

With the exception of the medio-lateral force component, the peak joint contact forces increased with increasing intensities in all joints and in all anatomical direction analysed (Fig 2). With regard to the phase of the squat cycle (eccentric, concentric), peak hip, tibiofemoral, and ankle joint contact forces were significantly higher in the concentric phase than in the eccentric phase. Patellofemoral joint contact forces did not show any significant difference related to the phase of the squat cycle.

The highest joint contact forces occurred at 90% 1-RM for each joint. Based on the mean values at 90% 1-RM across all participants, the peak resultant joint contact forces were 15.5 ± 3.0 BW, 23.2 ± 3.9 BW, 26.7 ± 4.3 BW, and 11.5 ± 2.2 BW for the hip, tibiofemoral, patellofemoral and ankle joint, respectively. Detailed values for joint contact forces in all anatomical directions, along with the exact statistical significance level (*p*), including the effect size (*η²*_*p*_) and the percentage change relative to 70% 1-RM of the corresponding phase, can be found in Table 2.

**Table 2:** Peak joint forces [BW in mean ± SD] and percentage change relative to 70% 1-RM of the corresponding phase in the hip, knee, and ankle joint with increasing intensity (70%, 75%, 80%, 85%, 90% of 1-RM).

Joint	Direction of force	70% 1-RM		75% 1-RM		80% 1-RM		85% 1-RM		90% 1-RM		Result for phase	Result for intensity
		Ecc	Con	Ecc	Con	Ecc	Con	Ecc	Con	Ecc	Con		
Hip	ap	−1.55 ± 0.34	−1.91 ± 0.42	−1.57 ± 0.38 **(1.3 ± 2.5%)**	−1.95 ± 0.40 **(2.1 ± 2.9%)**	−1.63 ± 0.44 **(5.2 ± 3.4%)**	−2.04 ± 0.46 **(6.8 ± 3.0%)**	−1.71 ± 0.40 **(10.3 ± 3.1%)**	−2.13 ± 0.45 **(11.5 ± 3.0%)**	−1.76 ± 0.42 **(13.5 ± 3.3%)**	−2.21 ± 0.48 **(15.7 ± 3.2%)**	Con > Ecc (absolute value) (p < 0.001*; η²p = 0.513)	Increases in negative direction with intensity (*p* < 0.001*; η²_p_ = 0.113)
	ml	0.30 ± 0.33	0.24 ± 0.34	0.31 ± 0.34 **(3.3 ± 15.8%)**	0.25 ± 0.34 **(4.2 ± 19.9%)**	0.27 ± 0.28 **(−10.0 ± 14.5%)**	0.26 ± 0.26 **(8.3 ± 17.0%)**	0.32 ± 0.39 **(6.7 ± 17.5%)**	0.34 ± 0.39 **(41.7 ± 22.2%)**	0.34 ± 0.37 **(13.3 ± 17.3%)**	0.36 ± 0.41 **(50.0 ± 22.6%)**	Con = Ecc (p = 0.632; η²_p_ = 0.003)	Increases with intensity (p = 0.004*; η²_p_ = 0.059)
	v	12.95 ± 3.20	13.63 ±3.07	13.37 ± 3.28 **(3.2 ± 4.2%)**	13.98 ± 3.06 **(2.6 ± 3.4**%)	13.63 ± 3.03 **(5.3 ± 4.0%)**	14.33 ± 2.98 **(5.1 ± 3.3%)**	14.37 ± 3.12 **(11.0 ± 4.0%)**	15.05 ± 3.05 **(10.4 ± 3.3%)**	14.65 ± 3.05 **(13.1 ± 4.1%)**	15.36 ± 2.97 **(12.7 ± 3.3%)**	Con > Ecc (*p* < 0.001*; η²_p_ = 0.122)	Increases with intensity (*p* < 0.001*; η²_p_ = 0.450)
	r	13.03 ± 3.21	13.70 ± 3.08	13.45 ± 3.29 **(3.2 ± 4.3%)**	14.06 ± 3.07 **(2.6 ± 3.3%)**	13.77 ± 3.03 **(5.7 ± 4.0%)**	14.42 ± 2.98 **(5.3 ± 3.2%)**	14.46 ± 3.12 **(11.0 ± 4.0%)**	15.16 ± 3.05 **(10.7 ± 3.2%)**	14.73 ± 3.05 **(13.1 ± 4.1%)**	15.46 ± 2.97 **(12.8 ± 3.3%)**	Con > Ecc (*p* < 0.001*; η²_p_ = 0.123)	Increases with intensity (*p* < 0.001*; η²_p_ = 0.449)
Tibio-femoral	ap	12.17 ± 2.38	12.79 ± 2.35	12.59 ± 2.35 **(3.5 ± 3.2%)**	13.02 ± 2.27 **(1.8 ± 2.7%)**	12.91 ± 2.46 **(6.1 ± 3.3%)**	13.35 ± 2.41 **(4.4 ± 2.8%)**	13.31 ± 2.42 **(9.4 ± 3.3%)**	13.69 ± 2.32 **(7.0 ± 2.8%)**	13.50 ± 2.50 **(10.9 ± 3.4%)**	13.82 ± 2.44 **(8.0 ± 2.9%)**	Con > Ecc (p < 0.001*; η²_p_ = 0.129)	Increases with intensity (p < 0.001*; η²_p_ = 0.512)
	ml	0.03 ± 0.09	0.08 ± 0.08	0.04 ± 0.10 **(33.3 ± 353%)**	0.08 ± 0.10 **(0.0 ± 176%)**	0.04 ± 0.09 **(33.3 ± 300%)**	0.10 ± 0.13 **(25.0 ± 229%)**	0.04 ± 0.08 **(33.3 ± 267%)**	0.10 ± 0.11 **(25.0 ± 190%)**	0.05 ± 0.11 **(66.7 ± 367%)**	0.12 ± 0.13 **(50.0 ± 229%)**	Con > Ecc (p = 0.005*; η²_p_ = 0.148)	Stays constant with intensity (p = 0.079; η²_p_ = 0.017)
	v	17.27 ± 2.77	17.72 ± 3.36	18.31 ± 2.97 **(6.0 ± 3.0%)**	18.23 ± 3.33 **(2.9 ± 3.4%)**	18.89 ± 2.99 **(9.4 ± 3.0%)**	18.84 ± 3.84 **(6.3 ± 5.5%)**	19.90 ± 3.40 **(15.2 ± 3.6%)**	19.28 ± 3.94 **(8.8 ± 6.2%)**	20.51 ± 3.49 **(18.8 ± 3.8%)**	19.85 ± 3.94 **(12.0 ± 6.4%)**	Con = Ecc (p = 0.396; η²_p_ = 0.005)	Increases with intensity (p < 0.001*; η²_p_ = 0.480)
	r	19.50 ± 3.10	20.74 ± 3.75	20.75 ± 3.34 **(6.4 ± 4.6%)**	21.41 ± 3.69 **(3.2 ± 4.3%)**	21.46 ± 3.33 **(9.5 ± 4.6%)**	22.13 ± 4.14 **(6.7 ± 6.1%)**	22.49 ± 3.68 **(15.3 ± 5.7%)**	22.59 ± 4.13 **(9.0 ± 6.5%)**	23.22 ± 3.86 **(19.3 ± 6.2%)**	23.13 ± 4.35 **(11.6 ± 6.8%)**	Con > Ecc (*p* = 0.021*; η²_p_ = 0.031)	Increases with intensity (p < 0.001*; η²_p_ = 0.531)
Patello-femoral	ap	18.92 ± 3.03	19.77 ± 3.51	20.11 ± 3.24 **(6.3 ± 3.5%)**	20.45 ± 3.51 **(3.4 ± 5.0%)**	20.72 ± 3.26 **(9.5 ± 3.6%)**	21.07 ± 3.90 **(6.6 ± 5.5%)**	21.74 ± 3.65 **(14.9 ± 4.3%)**	21.51 ± 3.90 **(8.8 ± 5.5%)**	22.40 ± 3.80 **(18.4 ± 4.6%)**	21.90 ± 4.15 **(10.8 ± 5.7%)**	Con = Ecc (*p* = 0.396; η²_p_ = 0.004)	Increases with intensity (p < 0.001*; η²_p_ = 0.533)
	ml	0.24 ±0.21	0.21 ± 0.22	0.26 ± 0.22 **(8.3 ± 12.7%)**	0.24 ± 0.19 **(14.3 ± 14.3%)**	0.28 ± 0.24 **(16.7 ± 14.2%)**	0.25 ± 0.22 **(19.0 ± 10.7%)**	0.30 ± 0.24 **(25 ± 14.2%)**	0.26 ± 0.21 **(23.8 ± 13.8%)**	0.29 ± 0.23 **(20.8 ± 13.5%)**	0.28 ± 0.23 **(33.3 ± 14.6%)**	Con = Ecc (p = 0.092; η²_p_ = 0.016)	Stays constant with intensity (p = 0.07; η²_p_ = 0.051)
	v	13.15 ±2.16	13.60 ± 2.14	13.86 ± 2.24 **(5.4 ± 3.0%)**	14.00 ± 2.29 **(2.9 ± 3.0%)**	14.14 ± 2.11 **(7.5 ± 2.8%)**	14.26 ± 2.28 **(4.9 ± 2.9%)**	14.71 ± 2.16 **(11.9 ± 2.7%)**	14.57 ± 2.22 **(7.1 ± 2.8%)**	15.02 ± 2.32 **(14.2 ± 2.9%)**	14.82 ± 2.31 **(8.9 ± 2.9%)**	Con = Ecc (p = 0.342; η²_p_ = 0.003)	Increases with intensity (p < 0.001*; η²_p_ = 0.591)
	r	22.80 ± 3.57	23.83 ± 3.95	24.17 ± 3.82 **(4.8 ± 4.4%)**	24.61 ± 4.00 **(3.3 ± 2.4%)**	24.88 ± 3.75 **(5.3 ± 3.8%)**	25.27 ± 4.35 **(6.0 ± 2.8%)**	26.07 ± 4.11 **(10.9 ± 4.4%)**	25.77 ± 4.31 **(8.1 ± 2.8%)**	26.73 ± 4.30 **(12.9 ± 4.0%)**	26.25 ± 4.64 **(10.2 ± 3.2%)**	Con = Ecc (*p* = 0.279; η²_p_ = 0.005)	Increases with intensity (p < 0.001*; η²_p_ = 0.559)
Ankle	ap	−4.55 ± 1.31	−4.90 ± 1.32	−4.73 ± 1.47 **(−4.0 ± 5.0%)**	−5.04 ± 1.43 **(−2.9 ± 4.5%)**	−4.87 ± 1.42 **(−7.0 ± 4.8%)**	−5.12 ± 1.36 **(−4.5 ± 4.2%)**	−5.03 ± 1.37 **(−10.5 ± 4.7%)**	−5.29 ± 1.33 **(−8.0 ± 4.1%)**	−5.19 ± 1.55 **(−14.1 ± 5.3%)**	−5.44 ± 1.48 **(−11.0 ± 4.4%)**	Con > Ecc (absolute value) (p < 0.001*; η²_p_ = 0.128)	Increases in negative direction with intensity (*p* = 0.001; η²_p_ = 0.270)
	ml	0.25 ± 0.14	0.31 ± 0.15	0.25 ± 0.14 **(0.0 ± 7.8%)**	0.28 ± 0.14 **(−9.7 ± 6.8%)**	0.26 ± 0.15 **(4.0 ± 8.0%)**	0.29 ± 0.16 **(−6.5 ± 7.4%)**	0.28 ± 0.14 **(12.0 ± 7.8%)**	0.32 ± 0.15 **(3.2 ± 6.8%)**	0.30 ± 0.16 **(20.0 ± 8.9%)**	0.35 ± 0.17 **(12.9 ± 7.2%)**	Con > Ecc (p = 0.002*; η²_p_ < 0.104)	Increases with intensity (p < 0.001*; η²_p_ = 0.089)
	v	8.41 ± 1.56	9.20 ± 1.71	8.82 ± 1.92 **(4.9 ± 6.0%)**	9.33 ± 1.78 **(1.4 ± 5.5%)**	9.03 ± 1.78 **(7.4 ± 5.5%)**	9.58 ± 1.72 **(4.1 ± 5.2%)**	9.26 ± 1.67 **(10.1 ± 5.2%)**	9.77 ± 1.48 **(6.2 ± 4.2%)**	9.57 ± 2.01 **(13.8 ± 6.0%)**	10.14 ± 1.92 **(10.2 ± 5.2%)**	Con > Ecc (p < 0.001*; η²_p_ = 0.135)	Increases with intensity (p < 0.001*; η²_p_ = 0.204)
	r	9.59 ± 1.93	10.44 ± 1.92	10.03 ± 2.33 **(4.6 ± 6.3%)**	10.61 ± 2.13 **(1.6 ± 5.4%)**	10.29 ± 2.16 **(7.3 ± 5.7%)**	10.82 ± 2.00 **(3.6 ± 5.1%)**	10.57 ± 2.03 **(10.2 ± 5.4%)**	11.13 ± 1.76 **(6.6 ± 4.3%)**	10.91 ± 2.43 **(13.7 ± 6.3%)**	11.48 ± 2.21 **(10.0 ± 5.3%)**	Con > Ecc (p < 0.001*; η²_p_ = 0.137)	Increases with intensity (p < 0.001*; η²_p_ = 0.239)

1-RM = 1-repetition maximum; ap = anterior-posterior; v = vertical; r = resultant; ecc = eccentric phase; con = concentric phase; * indicates significance.

### Joint contact force waveforms

**Fig 3:**
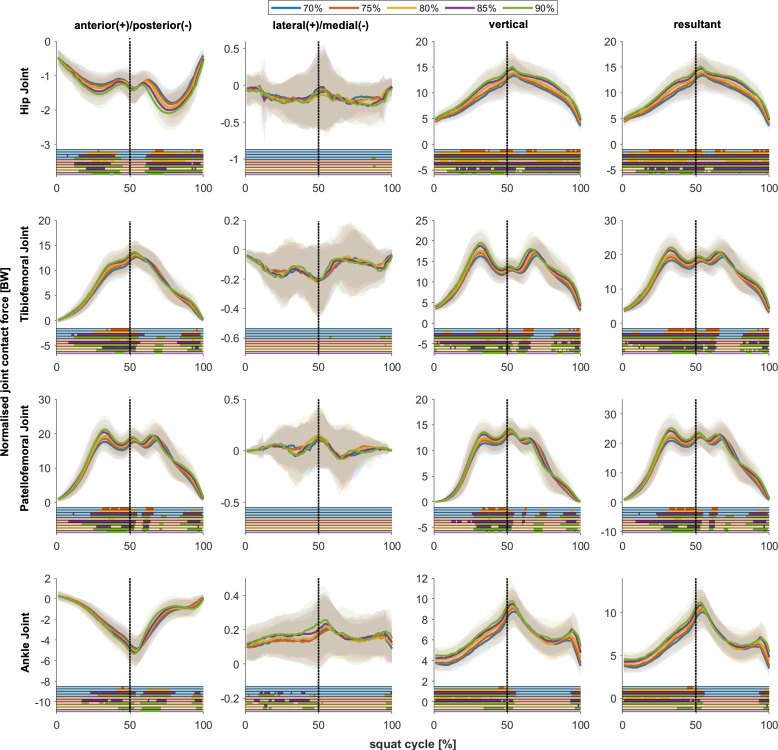
Joint contact forces throughout the squat cycle with different intensities. 0% and 100% of the squat cycle represent the respective upright position, and 50% denotes the lowest point of the sacrum, thus representing the deepest squat position. Solid lines in the figures represent the mean joint contact force across participants. Shaded areas represent ± one standard deviation. The lines below the figures indicate significant differences (SPM) during the squat cycle. The first four lines (light blue) represent significant differences between 70% 1-RM and 75% 1-RM, 70% 1-RM and 80% 1-RM, 70% 1-RM and 85% 1-RM, and 70% 1-RM and 90% 1-RM. The next three lines (orange) represent significant differences between 75% 1-RM and 80% 1-RM, 75% 1-RM and 85% 1-RM, and 75% 1-RM and 90% 1-RM. The following two lines (yellow) show significant difference between 80% 1-RM and 85% 1-RM, and 80% 1-RM and 90% 1-RM The last line (purple) shows significant differences between 85% 1-RM and 90% 1-RM.

Across a broad spectrum of the squat cycle, joint contact forces significantly changed with an increasing intensity in both the eccentric and concentric phases (Fig 3). However, there were also specific sections of the squat cycle where, despite increasing intensity, there was no significant change.

At the hip joint, the anterior-posterior force component was oriented in a posterior direction (posteriorly oriented force on femur in relation to pelvis) and demonstrated a rise in magnitude with increasing intensity. The medio-lateral forces, which showed only minimal changes with increasing intensities, were small compared to the other force components and had a medially oriented vector. A significant increase in vertical hip joint contact force was observed over almost the entire squat cycle with increasing intensity conditions. The resulting hip joint contact force exhibited a pattern comparable to that observed for the vertical forces.

In the tibiofemoral joint, the anterior-posterior, the vertical and the resultant force significantly increased with increasing intensities for most parts of the squat cycle. Similar to the hip joint, the media-lateral forces were relatively small compared to the other force components. In contrast to the anterior-posterior forces, which were highest around 50% of the squat cycle, the vertical and resultant forces showed two peaks – one in the middle of the eccentric phase and one in the middle of the concentric phase.

At the patellofemoral joint, the medio-lateral forces did not change across the intensity levels and were low compared to the other anatomical directions. In the remaining directions, significant differences between the intensity conditions were observed over parts of the squat cycle. However, the early eccentric phase and large parts of the concentric phase did not show any significant differences with increasing intensities.

The ankle joint exhibited a maximum contact force around 50% of the squat cycle. Significant differences with increasing intensity in all anatomical force directions, particularly in the eccentric phase of the squat, were observed. The medio-lateral, vertical and resultant ankle joint contact forces barely changed during the concentric phase with increasing intensities.

## Discussion

We showed that powerlifters are subjected to considerable joint contact forces during the squat, with some participants in our study experiencing joint contact forces beyond 20 times their BW. Our first hypothesis – peak joint contact forces increase with increasing intensities – was largely confirmed by our results. Over long periods of the squat cycle, joint contact forces increased in all joints and most anatomical force directions. Our second hypothesis – joint contact forces exhibit an increase throughout the squat cycle with increasing intensities – could only be partly confirmed. Interestingly, during substantial parts of the concentric phase, the tibiofemoral, patellofemoral and ankle joint contact forces showed no significant difference between intensity conditions. One possible explanation for this could be that muscle forces in this region did not significantly increase as intensity increased, as shown in S4 Fig in the Supporting Information. Since muscle forces play a crucial role in generating joint contact forces [39,40], constant muscle forces – as seen in some but not all muscles [2,45] – might explain the relatively constant joint contact forces during the concentric phase.

Peak hip and ankle joint contact forces were higher in the concentric phase than in the eccentric phase. This might be partly due to the muscle’s force-velocity properties. Muscle fibers can produce greater active tension at slower shortening velocities and at optimum lengths [46]. During the upward phase of the squat, which is usually performed with a very slow velocity, the muscles shorten at relatively low velocities – approaching near-isometric conditions – which enables the generation of very high muscle forces. These forces are necessary to overcome the combined effects of barbell weight, inertia, and gravity. In fact, it is precisely the necessity to actively counteract gravity during the upward phase – rather than “following it”, as in the downward phase – that increases the muscular demand, which in turn leads to slower contraction velocities, higher muscle forces and, consequently, elevated joint contact forces [39,40].

Both eccentric and concentric training improved function and reduced pain in patients with symptomatic knee osteoarthritis (OA) [47]. A systematic review showed that eccentric training may offer potential benefits in musculoskeletal rehabilitation [48]. We found lower hip and ankle joint contact forces in the eccentric compared to the concentric phase. However, it is important to note that the differences of the resultant joint contact forces between the eccentric and concentric phase were consistently below 10% BW (S1 Table). While these differences are small, they could still be of potential relevance in specific contexts, such as injury prevention and rehabilitation.

Comparing our findings with previous research is difficult due to the absence of studies with elite powerlifters squatting with high barbell loads. In the field of performance-oriented squats, Salem et al. examined the relationship between joint contact forces and squat depth, which showed that patellofemoral joint contact forces only increased slightly from 6.0 BW to 7.1 BW with higher knee flexion [15]. It is important to note that although the participants in the study conducted by Salem et al. performed squats with 85% 1-RM, the additional load utilized was on average only 53 ± 12 kg [15], which is a lot lower compared to our participants with an average barbell load of 180 ± 61 kg at the 90% 1-RM condition and likely explains the higher forces in our study (detailed information on the 1-RM values of our participants is available in S2 Table of the Supporting Information).

In vivo measured joint contact forces using instrumented endoprostheses ranged from 2.1 to 6.5 BW in the patellofemoral joint [49] and from 1.6 to 5.4 BW in the tibiofemoral joint [49]. In the hip joint, forces of approximately 2 BW were observed during squats without additional loads [50]. Musculoskeletal simulations of body weight squat estimated 2.5 ± 0.3 BW for the tibiofemoral joint [17] and 2.5 to 3.0 BW for the hip joint [18]. When considering that all of the aforementioned values represent squats without additional loads, that our athletes lifted far more than twice their BW as additional load on the barbell in a deep squat movement, and that our estimated joint contact force waveforms exhibit a similar shape to those measured by instrumented endoprostheses, we are confident that our values are reasonable.

A factor not considered in this study, but which could have an influence on joint contact forces, is the contact between the calf and the thigh. This contact can result in substantial force transfer during squats [51]. Both compression and shear forces in the knee joint are reduced by the thigh-calf contact during high knee flexion [52,53]. This effect is more pronounced the greater the sum of the thigh and calf circumferences [53]. As our participants were elite powerlifters, this effect is likely to be important due to their large muscle mass and should be taken into account in future studies as the influence on muscle and joint contact forces could be substantial. The use of knee sleeves worn by all participants could have further enhanced this effect [54]. Similarly, contact between the trunk and thighs and the wearing of a belt can also lead to changes in joint contact forces. Unfortunately, there are no studies on this aspect yet.

The individual morphological characteristics may also influence joint contact forces and the resulting stress on the joint structures. It is important to note that the joint contact forces represent only one factor among many that contribute to the overall stress experienced by joint structures. The dimensions and mechanical characteristics of the joint surfaces and surrounding tissue influence the stress on the anatomical structures such as cartilage and ligaments. To illustrate, identical joint contact forces but different femoral head sizes can result in different hip cartilage stress. Hence, the individual-specific bony geometry [55,56], additionally to the movement execution of the squat [49,57], determine the stress placed on the involved anatomical structures.

Heavy resistance training can elicit both adaptive and maladaptive responses in articular cartilage. On the one hand, high mechanical loading may promote functional cartilage adaptation, as elite weightlifters show greater knee cartilage thickness than non-athletes, suggesting post-maturation hypertrophy [58,59]. Moderate exercise can also enhance cartilage composition [60]. Consequently, long-term supervised strength training appears to be safe for cartilage and may be protective against OA [61]. Animal studies further indicate that specific high-impact stimuli, like plyometric training, can increase cartilage thickness [62].

Conversely, excessive or prolonged joint loading may contribute to cartilage degeneration. Epidemiological data show elevated rates of knee OA, particularly in the patellofemoral joint, among former strength athletes [63]. A recent systematic review further links weightlifting and other high-impact sports to molecular and structural cartilage changes conducive to OA [64]. Elevated body mass may further exacerbate joint stress, partly explaining the increased OA risk in weightlifters [63]. These findings support the concept of a loading threshold for cartilage homeostasis, beyond which catabolic processes may be triggered [64]. The joint contact force data presented in this study provide context for these findings, although the determination of individualised load thresholds remains a topic for future investigation.

In light of the capacity for both adaptive and maladaptive cartilage responses to heavy resistance training, appropriate load management is essential to maximise benefits and minimise risks in strength sports. While mechanical loading can enhance cartilage properties, avoiding chronic overload is essential. Principles such as gradual progression, adequate recovery, and targeted injury prevention are crucial to support joint health. Hence, with well-designed training protocols, cartilage adaptation may be facilitated, while unrelenting stress may lead to degeneration. Although deep squats generate high joint contact forces, they are not inherently detrimental when executed with proper technique and progressive loading [65]. A nuanced understanding of joint mechanics and periodisation strategies – including strategic deload phases [66,67] – is critical to balancing performance goals with joint preservation.

While musculoskeletal simulations using Static Optimization offer computational efficiency and simplicity, it has inherent limitations that can affect the accuracy of joint contact force predictions. Static Optimization operates under the assumption that muscle forces can be determined independently at each time point, disregarding the temporal dynamics of muscle activation and contraction [68]. Further, it typically aims to minimise a cost function, such as the sum of squared muscle activations, which may lead to an underestimation of muscle co-contraction. This can result in inaccuracies in joint contact force estimations, particularly in movements where co-contraction plays a role [69]. As illustrated in the validation plot (S3 Fig in the Supporting Information), the activation waveforms estimated via Static Optimization showed a good agreement with surface EMG signals across most muscles. However, activation of the rectus femoris was virtually absent in the simulation, despite clear EMG activity in this muscle. This discrepancy is a known and well-documented limitation of Static Optimization approaches, particularly for biarticular muscles such as the rectus femoris [70–72]. The minimisation-based cost function used in Static Optimization tends to reduce activations that generate opposing joint torques, which can result in physiologically unrealistic suppression of such muscles during tasks involving simultaneous hip and knee extension. Incorporating participant-specific electromyography data in the simulations might enhance the accuracy of muscle force estimations.

This study had some limitations. First, participants performed only one squat per intensity level. Given their extensive training and the large sample size, we consider this sufficient to test our hypotheses. Moreover, the inclusion of additional repetitions at the high intensity levels would have introduced a bias due to fatigue. Secondly, the influence of individual differences in bone structure, muscle attachment points and, consequently, moment arms [56] and individual muscle coordination strategies (i.e., EMG-informed neuromusculoskeletal modelling) [73–75] was not considered. However, previous studies have shown that muscle forces – and consequently, joint contact forces – are more significantly affected by movement patterns than by bone morphology [76], while bone morphology itself has a greater effect than muscle coordination [77]. Therefore, the most critical factors were considered in our study. Likewise, applying finite element (FE) modelling would enable the evaluation of tissue-level loads (cartilage stress, tendon strain, subchondral bone pressure). For example, FE analyses of deep squatting reveal markedly elevated articular cartilage stresses relative to neutral standing [78], a finding that underscores the need to assess such high-load scenarios to understand potential tissue damage and long-term joint adaptation. Future research should include advanced EMG- and medical imaging-informed musculoskeletal simulations to enhance participant-specific estimates of joint contact forces as well as FE simulations to quantify tissue-level loads in elite powerlifters. Thirdly, the validation of the simulation was based on in vivo data from individuals with instrumented joint replacements [41]. These datasets originate from an older and less athletic population with substantially lower external loads than those observed in our elite powerlifters. Moreover, Schellenberg et al. [79] showed that musculoskeletal models can overestimate tibiofemoral joint contact forces by up to 60% at deep knee flexion angles (≥80°), which are common in powerlifting squats. However, their findings refer only to models that were available at the time of their study. In our work, we used a musculoskeletal model [28] that was published after Schellenberg et al. [79] and specifically designed to improve accuracy in deep flexion. Although direct in vivo validation in athletic populations remains challenging, this more recent model implementation may reduce the overestimation bias in high flexion angles. Nevertheless, the absolute joint contact force values – particularly in deep flexion – should still be interpreted with caution.

## Conclusion

The current study demonstrated that the joint contact forces of the hip, tibiofemoral, patellofemoral and ankle joint increased in squats with increasing intensities, yet not during all periods of the squat cycle. Joint contact forces in elite powerlifters reached more than 20 times their BW. These results have several practical implications. From a performance perspective, our study highlights the importance of load tolerance in the hip and knee joints. Given the high joint contact forces observed, coaches should consider structured periodisation and deloading strategies to balance adaptation with joint preservation. Variations in squat technique may also help modulate joint stress while maintaining stimulus. Furthermore, understanding joint contact force profiles can inform rehabilitation protocols to minimise joint loads. While the study focused on elite athletes, the insights may also apply to well-trained individuals or clinical populations seeking to build strength with preserving joint integrity over time. Knowledge of joint contact forces during squats can help to optimise load progression and movement selection, contributing to long-term joint health and injury prevention in both competitive and general strength training settings.

## Supporting information

S1 Fig Validation of simulations. Hip and knee resultant joint contact force waveforms of squats (body weight only, 15 kg bar, 35 kg total) of one participant were comparable to those obtained from a participant with an instrumented hip implant and another participant with an instrumented knee implant from the Orthoload database. The shape of the Orthoload waveforms were similar to the waveforms obtained from our athlete. Visual comparison between the hip joint contact forces from the participants in our study with those found on the Orthoload database showed a reasonable agreement with our simulations. It should be noted that the hip and knee joint contact force waveforms from the Orthoload database each were from one participant. Differences between our results and the values from Orthoload might be caused by a combination of differences in hip and knee kinematics, bone and muscle morphology, movement execution technique and velocities, additionally to the different methods to obtain the joint contact forces (simulations versus in-vivo measurement).
(DOCX)

S2 Fig Validation of simulations with Perrone et al. (2023). Hip resultant joint contact force waveforms of squats (body weight only) of one participant were comparable to single-legged squats obtained from the results of Perrone et al. (2023). The shape of this study’s waveforms was similar to the waveforms obtained from our athlete. The differences observed between our absolute values and the results reported by Perrone et al. (2023) may be attributable to several factors: firstly, the execution of single-legged half squats in the study by Perrone et al. (2023) instead of double-legged deep squats as in the present study. Secondly, the differences in the model employed, the movement execution technique, and the velocities may have contributed to the discrepancies in results.
(DOCX)

S3 Fig Comparison of muscle activations estimated via static optimization and measured using surface electromyography during the squat movement. 0% and 100% of the squat cycle represent the upright standing position, while 50% corresponds to the lowest vertical position of the sacrum and thus the deepest point of the squat. Blue lines indicate muscle activations estimated via Static Optimization, and red lines represent muscle activations measured using surface electromyography (EMG). EMG signals were normalized to the peak activation value obtained from Static Optimization for comparability of waveform profiles.
(DOCX)

S4 Fig Change of muscle forces throughout the squat cycle with different intensities. 0% and 100% of the squat cycle represent the respective upright position, and 50% denotes the lowest point of the sacrum, thus representing the deepest squat position. Solid lines in the figures represent the mean joint contact force across participants. Shaded areas represent ± one standard deviation. The lines below the figures indicate significant differences (SPM) during the squat cycle. The first four lines (light blue) represent significant differences between 70% 1-RM and 75% 1-RM, 70% 1-RM and 80% 1-RM, 70% 1-RM and 85% 1-RM, and 70% 1-RM and 90% 1-RM. The next three lines (orange) represent significant differences between 75% 1-RM and 80% 1-RM, 75% 1-RM and 85% 1-RM, and 75% 1-RM and 90% 1-RM. The following two lines (yellow) show significant difference between 80% 1-RM and 85% 1-RM, and 80% 1-RM and 90% 1-RM The last line (purple) shows significant differences between 85% 1-RM and 90% 1-RM.
(DOCX)

S5 Fig Muscle segmentation using 3D slicer. a) Manual annotation of the target structures performed in the “Segment Editor” using the “Paint” function. At least ten slices per muscle were marked in each axis to provide a sufficient basis for the interpolation of the intermediate layers. b) “Fill between Slices” function was used to create a continuous segmentation. c) Where interpolation errors were noted, further slices were incorporated until a complete and anatomically precise segmentation was realised.
(DOCX)

S1 Table Percentage differences between the eccentric and concentric joint contact force during squats with increasing intensity (70%, 75%, 80%, 85%, 90% of 1-RM). 1-RM = 1-repetition maximum.
(DOCX)

S2 Table 1-RM values and gender of the participants. 1-RM = 1-repetition maximum.
(DOCX)

## References

[1] Goodman WW, Helms E, Graham DF. Individual muscle contributions to the acceleration of the center of mass during the barbell back squat in trained female subjects. J Strength Cond Res. 2023;37(10):1947–54. doi: 10.1519/JSC.0000000000004506 37556813

[2] Kipp K, Kim H, Wolf WI. Muscle forces during the squat, split squat, and step-up across a range of external loads in college-aged men. J Strength Cond Res. 2022;36(2):314–23. doi: 10.1519/JSC.0000000000003688 32569122

[3] Larsen S, de Zee M, Kristiansen EL, van den Tillaar R. A biomechanical comparison between a high and low barbell placement on net joint moments, kinematics, muscle forces, and muscle-specific moments in 3 repetition maximum back squats. J Strength Cond Res. 2024;38(7):1221–30. doi: 10.1519/JSC.0000000000004790 38900172

[4] Bryanton MA, Carey J, Kennedy MD, Chiu LZF. Quadriceps effort during squat exercise depends on hip extensor muscle strategy. Sports Biomech. 2015;14(1):122–38. 25895990 10.1080/14763141.2015.1024716

[5] Kushner AM, Brent JL, Schoenfeld BJ, Hugentobler J, Lloyd RS, Vermeil A, et al. The back squat part 2: targeted training techniques to correct functional deficits and technical factors that limit performance. Strength Cond J. 2015;37(2):13–60. doi: 10.1519/SSC.0000000000000130 26823657 PMC4725067

[6] Myer GD, Kushner AM, Brent JL, Schoenfeld BJ, Hugentobler J, Lloyd RS, et al. The back squat: a proposed assessment of functional deficits and technical factors that limit performance. Strength Cond J. 2014;36(6):4–27. doi: 10.1519/SSC.0000000000000103 25506270 PMC4262933

[7] Schoenfeld BJ. Squatting kinematics and kinetics and their application to exercise performance. J Strength Cond Res. 2010;24(12):3497–506. doi: 10.1519/JSC.0b013e3181bac2d7 20182386

[8] Aube 8 D, Wahi T, Rauch J, Anand A, Barakat C, Pearson J, et al. Progressive resistance training volume: effects on muscle thickness, mass, and strength adaptations in resistance-trained individuals. J Strength Cond Res. 2022;36(3):600–7. 32058362 10.1519/JSC.0000000000003524

[9] Schoenfeld BJ, Contreras B, Vigotsky AD, Peterson M. Differential effects of heavy versus moderate loads on measures of strength and hypertrophy in resistance-trained men. J Sports Sci Med. 2016;15(4):715–22. 27928218 PMC5131226

[10] Asayama A, Tateuchi H, Yamagata M, Ichihashi N. Influence of stance width and toe direction on medial knee contact force during bodyweight squats. J Biomech. 2021;129:110824. doi: 10.1016/j.jbiomech.2021.110824 34717162

[11] Chauhan SS. Kinematic and kinetic analysis of knee joint during squatting. IOP Conf Ser Mater Sci Eng. 2019;691(1):012020.

[12] Kothurkar R, Lekurwale R, Gad M, Rathod CM. Estimation and comparison of knee joint contact forces during heel contact and heel rise deep squatting. Indian J Orthop. 2022;57(2):310–8. doi: 10.1007/s43465-022-00798-y 36777124 PMC9880086

[13] Escamilla RF. Knee biomechanics of the dynamic squat exercise. Med Sci Sports Exerc. 2001;33(1):127–41. doi: 10.1097/00005768-200101000-00020 11194098

[14] Hartmann H, Wirth K, Klusemann M. Analysis of the load on the knee joint and vertebral column with changes in squatting depth and weight load. Sports Med. 2013;43(10):993–1008. 23821469 10.1007/s40279-013-0073-6

[15] Salem GJ, Powers CM. Patellofemoral joint kinetics during squatting in collegiate women athletes. Clin Biomech. 2001;16(5):424–30. 10.1016/s0268-0033(01)00017-1 11390050

[16] Perrone M, Guidetti M, Galli M, Nho SJ, Wimmer MA, Malloy P. Hip joint contact forces are lower in people with femoroacetabular impingement syndrome during squat tasks. J Orthop Res. 2024;42(5):1045–53. doi: 10.1002/jor.25744 38032092

[17] Lu Z, Li X, Rong M, Baker JS, Gu Y. Effect of rearfoot valgus on biomechanics during barbell squatting: a study based on OpenSim musculoskeletal modeling. Front Neurorobot. 2022;16:832005. doi: 10.3389/fnbot.2022.832005 36017416 PMC9396136

[18] Catelli DS, Kowalski E, Beaulé PE, Lamontagne M. Muscle and hip contact forces in asymptomatic men with cam morphology during deep squat. Front Sports Act Living. 2021;3:716626. doi: 10.3389/fspor.2021.716626 34568821 PMC8458768

[19] Meireles S, De Groote F, Reeves ND, Verschueren S, Maganaris C, Luyten F, et al. Knee contact forces are not altered in early knee osteoarthritis. Gait Posture. 2016;45:115–20. doi: 10.1016/j.gaitpost.2016.01.016 26979892

[20] Moyer RF, Ratneswaran A, Beier F, Birmingham TB. Osteoarthritis year in review 2014: mechanics--basic and clinical studies in osteoarthritis. Osteoarthritis Cartilage. 2014;22(12):1989–2002. doi: 10.1016/j.joca.2014.06.034 25456294

[21] Willinger L, Foehr P, Achtnich A, Forkel P, Voss A, Liska F, et al. Effect of lower limb alignment in medial meniscus-deficient knees on tibiofemoral contact pressure. Orthop J Sports Med. 2019;7(2):2325967118824611. doi: 10.1177/2325967118824611 30800688 PMC6378645

[22] Castro-Viñuelas R, Viudes-Sarrión N, Rojo-García A, Monteagudo S, Lories R, Jonkers I. Mechanical loading rescues mechanoresponsiveness in a human osteoarthritis explant model despite Wnt activation. Osteoarthritis Cartilage. 2024;S1063-4584(24)01015-X. 10.1016/j.joca.2024.02.945 38494072

[23] Yack JH, Washco LA, Whieldon T. compressive forces as a limiting factor of anterior tibial translation in the ACL-deficient knee. Clin J Sport Med. 1994;4(4):233–9.

[24] International Powerlifting Federation. International Powerlifting Federation [Online]; 2024. [cited 2024 Aug 15]. Available from: https://www.powerlifting.sport/fileadmin/ipf/data/rules/approved-list/Approved__List_2023-2026_V2_01-11-2023.pdf

[25] International Powerlifting Federation. International Powerlifting Federation [Online]; 2024 [cited 2024 Aug 15]. Available from: https://www.powerlifting.sport/rules/codes/info/technical-rules

[26] Fedorov A, Beichel R, Kalpathy-Cramer J, Finet J, Fillion-Robin J-C, Pujol S, et al. 3D Slicer as an image computing platform for the Quantitative Imaging Network. Magn Reson Imaging. 2012;30(9):1323–41. doi: 10.1016/j.mri.2012.05.001 22770690 PMC3466397

[27] Delp SL, Anderson FC, Arnold AS, Loan P, Habib A, John CT, et al. OpenSim: open-source software to create and analyze dynamic simulations of movement. IEEE Trans Biomed Eng. 2007;54(11):1940–50. doi: 10.1109/TBME.2007.901024 18018689

[28] Catelli DS, Wesseling M, Jonkers I, Lamontagne M. A musculoskeletal model customized for squatting task. Comput Methods Biomech Biomed Engin. 2019;22(1):21–4. doi: 10.1080/10255842.2018.1523396 30398067

[29] Kainz H, Hoang HX, Stockton C, Boyd RR, Lloyd DG, Carty CP. Accuracy and reliability of marker-based approaches to scale the pelvis, thigh, and shank segments in musculoskeletal models. J Appl Biomech. 2017:354–60. 28290736 10.1123/jab.2016-0282

[30] Kainz H, Goudriaan M, Falisse A, Huenaerts C, Desloovere K, De Groote F, et al. The influence of maximum isometric muscle force scaling on estimated muscle forces from musculoskeletal models of children with cerebral palsy. Gait Posture. 2018;65:213–20. doi: 10.1016/j.gaitpost.2018.07.172 30558934

[31] van der Krogt MM, Bar-On L, Kindt T, Desloovere K, Harlaar J. Neuro-musculoskeletal simulation of instrumented contracture and spasticity assessment in children with cerebral palsy. J Neuroeng Rehabil. 2016;13(1):64. doi: 10.1186/s12984-016-0170-5 27423898 PMC4947289

[32] Hicks JL, Uchida TK, Seth A, Rajagopal A, Delp SL. Is my model good enough? Best practices for verification and validation of musculoskeletal models and simulations of movement. J Biomech Eng. 2015;137(2):020905. doi: 10.1115/1.4029304 25474098 PMC4321112

[33] Handsfield GG, Meyer CH, Hart JM, Abel MF, Blemker SS. Relationships of 35 lower limb muscles to height and body mass quantified using MRI. J Biomech. 2014;47(3):631–8. doi: 10.1016/j.jbiomech.2013.12.002 24368144

[34] Modenese L, Montefiori E, Wang A, Wesarg S, Viceconti M, Mazzà C. Investigation of the dependence of joint contact forces on musculotendon parameters using a codified workflow for image-based modelling. J Biomech. 2018;37:108–18. 10.1016/j.jbiomech.2018.03.039 29673935

[35] Buchanan TS. Evidence that maximum muscle stress is not a constant: differences in specific tension in elbow flexors and extensors. Med Eng Phys. 1995;17(7):529–36. doi: 10.1016/1350-4533(95)00005-8 7489126

[36] Ikegawa S, Funato K, Tsunoda N, Kanehisa H, Fukunaga T, Kawakami Y. Muscle force per cross-sectional area is inversely related with pennation angle in strength trained athletes. J Strength Cond Res. 2008;22(1):128–31. doi: 10.1519/JSC.0b013e31815f2fd3 18296965

[37] Powell PL, Roy RR, Kanim P, Bello MA, Edgerton VR. Predictability of skeletal muscle tension from architectural determinations in guinea pig hindlimbs. J Appl Physiol Respir Environ Exerc Physiol. 1984;57(6):1715–21. doi: 10.1152/jappl.1984.57.6.1715 6511546

[38] Lieber RL, Fridén J. Functional and clinical significance of skeletal muscle architecture. Muscle Nerve. 2000;23(11):1647–66. doi: 10.1002/1097-4598(200011)23:11<1647::aid-mus1>3.0.co;2-m 11054744

[39] Correa TA, Crossley KM, Kim HJ, Pandy MG. Contributions of individual muscles to hip joint contact force in normal walking. J Biomech. 2010;43(8):1618–22. doi: 10.1016/j.jbiomech.2010.02.008 20176362

[40] Sasaki K, Neptune RR. Individual muscle contributions to the axial knee joint contact force during normal walking. J Biomech. 2010;43(14):2780–4. doi: 10.1016/j.jbiomech.2010.06.011 20655046 PMC2963724

[41] Bergmann G. Database orthoload; 2008. [cited 2024 Dec 13]. Available from: https://orthoload.com/database/

[42] Pataky TC. Generalized n-dimensional biomechanical field analysis using statistical parametric mapping. J Biomech. 2010;43(10):1976–82. doi: 10.1016/j.jbiomech.2010.03.008 20434726

[43] SPM1D.org, “package for one-dimensional Statistical Parametric Mapping”; 2022. [Online]. Available from: https://spm1d.org

[44] Kang H. Sample size determination and power analysis using the G*Power software. J Educ Eval Health Prof. 2021;18(17):Epub 2021. 10.3352/jeehp.2021.18.17 PMC8441096 34325496

[45] Pürzel A, Kaufmann P, Koller W, Pöhlmann L, Baca A, Kainz H. Muscle force dynamics across increasing squat intensity conditions in elite powerlifters. Scand J Med Sci Sports. 2025;35(5):e70058. doi: 10.1111/sms.70058 40275729

[46] Alcazar J, Csapo R, Ara I, Alegre LM. On the shape of the force-velocity relationship in skeletal muscles: the linear, the hyperbolic, and the double-hyperbolic. Front Physiol. 2019;10:769. doi: 10.3389/fphys.2019.00769 31275173 PMC6593051

[47] Trojani M-C, Chorin F, Gerus P, Breuil V, Michel C, Guis S, et al. Concentric or eccentric physical activity for patients with symptomatic osteoarthritis of the knee: a randomized prospective study. Ther Adv Musculoskelet Dis. 2022;14:1759720X221102805. doi: 10.1177/1759720X221102805 35832351 PMC9272179

[48] Kristensen J, Franklyn-Miller A. Resistance training in musculoskeletal rehabilitation: a systematic review. Br J Sports Med. 2012;46(10):719–26. doi: 10.1136/bjsm.2010.079376 21791457

[49] Innocenti B, Pianigiani S, Labey L, Victor J, Bellemans J. Contact forces in several TKA designs during squatting: a numerical sensitivity analysis. J Biomech. 2011;44(8):1573–81. doi: 10.1016/j.jbiomech.2011.02.081 21435645

[50] Stansfield BW, Nicol AC, Paul JP, Kelly IG, Graichen F, Bergmann G. Direct comparison of calculated hip joint contact forces with those measured using instrumented implants. An evaluation of a three-dimensional mathematical model of the lower limb. J Biomech. 2003;36(7):929–36. doi: 10.1016/s0021-9290(03)00072-1 12757801

[51] Kingston DC, Acker SM. Thigh-calf contact parameters for six high knee flexion postures: onset, maximum angle, total force, contact area, and center of force. J Biomech. 2018;67:46–54. doi: 10.1016/j.jbiomech.2017.11.022 29248190

[52] Wu JZ, Sinsel EW, Carey RE, Zheng L, Warren CM, Breloff SP. Biomechanical modeling of deep squatting: effects of the interface contact between posterior thigh and shank. J Biomech. 2019;96:109333. doi: 10.1016/j.jbiomech.2019.109333 31558308

[53] Zelle J, Barink M, De Waal Malefijt M, Verdonschot N. Thigh-calf contact: does it affect the loading of the knee in the high-flexion range? J Biomech. 2009;42(5):587–93. doi: 10.1016/j.jbiomech.2008.12.015 19200996

[54] Dooley E, Carr J, Carson E, Russell S. The effects of knee support on the sagittal lower-body joint kinematics and kinetics of deep squats. J Biomech. 2019;82:164–70. doi: 10.1016/j.jbiomech.2018.10.024 30446216

[55] Anderson AE, Ellis BJ, Maas SA, Weiss JA. Effects of idealized joint geometry on finite element predictions of cartilage contact stresses in the hip. J Biomech. 2010;43(7):1351–7. doi: 10.1016/j.jbiomech.2010.01.010 20176359 PMC2857573

[56] Kainz H, Mindler GT, Kranzl A. Influence of femoral anteversion angle and neck-shaft angle on muscle forces and joint loading during walking. PLoS One. 2023;18(10):e0291458. doi: 10.1371/journal.pone.0291458 37824447 PMC10569567

[57] Hale R, Green J, Hausselle J, Saxby D, Gonzalez RV. Quantified in vitro tibiofemoral contact during bodyweight back squats. J Biomech. 2018;79:21–30. doi: 10.1016/j.jbiomech.2018.07.002 30197151

[58] Grzelak P, Domzalski M, Majos A, Podgórski M, Stefanczyk L, Krochmalski M, et al. Thickening of the knee joint cartilage in elite weightlifters as a potential adaptation mechanism. Clin Anat. 2014;27(6):920–8. doi: 10.1002/ca.22393 24648385

[59] Gratzke C, Hudelmaier M, Hitzl W, Glaser C, Eckstein F. Knee cartilage morphologic characteristics and muscle status of professional weight lifters and sprinters: a magnetic resonance imaging study. Am J Sports Med. 2007;35(8):1346–53. doi: 10.1177/0363546507299746 17376857

[60] Roos EM, Dahlberg L. Positive effects of moderate exercise on glycosaminoglycan content in knee cartilage: a four-month, randomized, controlled trial in patients at risk of osteoarthritis. Arthritis Rheum. 2005;52(11):3507–14. doi: 10.1002/art.21415 16258919

[61] Lo GH, Richard MJ, McAlindon TE, Kriska AM, Price LL, Rockette-Wagner B, et al. Strength training is associated with less knee osteoarthritis: data from the osteoarthritis initiative. Arthritis Rheumatol. 2024;76(3):377–83. doi: 10.1002/art.42732 37870119 PMC10922276

[62] Chiaberge M, Thottappillil N, Liphardt A-M, Furlanetto A, Odell D, Wang C, et al. Plyometric training increases thickness and volume of knee articular cartilage in mice. NPJ Microgravity. 2025;11(1):5. doi: 10.1038/s41526-025-00458-z 39948108 PMC11825961

[63] Kujala UM, Kettunen J, Paananen H, Aalto T, Battié MC, Impivaara O, et al. Knee osteoarthritis in former runners, soccer players, weight lifters, and shooters. Arthritis Rheum. 1995;38(4):539–46. doi: 10.1002/art.1780380413 7718008

[64] Trovato B, Petrigna L, Sortino M, Roggio F, Musumeci G. The influence of different sports on cartilage adaptations: a systematic review. Heliyon. 2023;9(3):e14136. doi: 10.1016/j.heliyon.2023.e14136 36923870 PMC10009456

[65] Rojas-Jaramillo A, Cuervo-Arango DA, Quintero JD, Ascuntar-Viteri JD, Acosta-Arroyave N, Ribas-Serna J, et al. Impact of the deep squat on articular knee joint structures, friend or enemy? A scoping review. Front Sports Act Living. 2024;6:1477796. doi: 10.3389/fspor.2024.1477796 39640505 PMC11618833

[66] Bell L, Nolan D, Immonen V, Helms E, Dallamore J, Wolf M, et al. “You can’t shoot another bullet until you’ve reloaded the gun”: coaches’ perceptions, practices and experiences of deloading in strength and physique sports. Front Sports Act Living. 2022;4:1073223. doi: 10.3389/fspor.2022.1073223 36619355 PMC9811819

[67] Bell L, Strafford BW, Coleman M, Androulakis Korakakis P, Nolan D. Integrating deloading into strength and physique sports training programmes: an international delphi consensus approach. Sports Med Open. 2023;9(1):87. doi: 10.1186/s40798-023-00633-0 37730925 PMC10511399

[68] Prilutsky BI, Zatsiorsky VM. Optimization-based models of muscle coordination. Exerc Sport Sci Rev. 2002;30(1):32–8. doi: 10.1097/00003677-200201000-00007 11800497 PMC2821033

[69] Morrow MM, Rankin JW, Neptune RR, Kaufman KR. A comparison of static and dynamic optimization muscle force predictions during wheelchair propulsion. J Biomech. 2014;47(14):3459–65. doi: 10.1016/j.jbiomech.2014.09.013 25282075 PMC4254308

[70] Imani Nejad Z, Khalili K, Hosseini Nasab SH, Schütz P, Damm P, Trepczynski A, et al. The capacity of generic musculoskeletal simulations to predict knee joint loading using the CAMS-knee datasets. Ann Biomed Eng. 2020;48(4):1430–40. doi: 10.1007/s10439-020-02465-5 32002734 PMC7089909

[71] Sjöberg M, Berg HE, Norrbrand L, Andersen MS, Gutierrez-Farewik EM, Sundblad P, et al. Comparison of joint and muscle biomechanics in maximal flywheel squat and leg press. Front Sports Act Living. 2021;3:686335. doi: 10.3389/fspor.2021.686335 34423289 PMC8374053

[72] Higa M, Endo Y, Nakagawa Y. Force estimations and theoretical calculations for the biarticular muscles during squatting. JBSE. 2022;17(3):22-00060-22–00060. doi: 10.1299/jbse.22-00060

[73] Kainz H, Falisse A, Pizzolato C. Neuromusculoskeletal modeling in health and disease. Braz J Mot Behav. 2024;18(1).

[74] Kainz H, Jonkers I. Imaging-based musculoskeletal models alter muscle and joint contact forces but do not improve the agreement with experimentally measured electromyography signals in children with cerebral palsy. Gait Posture. 2023;100:91–5. doi: 10.1016/j.gaitpost.2022.11.019 36502666

[75] Pizzolato C, Lloyd DG, Sartori M, Ceseracciu E, Besier TF, Fregly BJ, et al. CEINMS: a toolbox to investigate the influence of different neural control solutions on the prediction of muscle excitation and joint moments during dynamic motor tasks. J Biomech. 2015;48(14):3929–36. doi: 10.1016/j.jbiomech.2015.09.021 26522621 PMC4655131

[76] Koller W, Baca A, Kainz H. The gait pattern and not the femoral morphology is the main contributor to asymmetric hip joint loading. PLoS One. 2023;18(9):e0291789. doi: 10.1371/journal.pone.0291789 37751435 PMC10522038

[77] Kainz H, Wesseling M, Jonkers I. Generic scaled versus subject-specific models for the calculation of musculoskeletal loading in cerebral palsy gait: effect of personalized musculoskeletal geometry outweighs the effect of personalized neural control. Clin Biomech (Bristol). 2021;87:105402. doi: 10.1016/j.clinbiomech.2021.105402 34098149

[78] Kothurkar R, Lekurwale R, Gad M, Rathod CM. Finite element analysis of a healthy knee joint at deep squatting for the study of tibiofemoral and patellofemoral contact. J Orthop. 2023;40:7–16. doi: 10.1016/j.jor.2023.04.016 37143926 PMC10151221

[79] Schellenberg F, Taylor WR, Trepczynski A, List R, Kutzner I, Schütz P, et al. Evaluation of the accuracy of musculoskeletal simulation during squats by means of instrumented knee prostheses. Med Eng Phys. 2018;61:95–9. doi: 10.1016/j.medengphy.2018.09.004 30282587

