# Supplementary material for: Biomechanical analysis of hip, knee, and ankle joint contact forces during squats in elite powerlifters
Source: PLoS One. 2025 Jul 24;20(7):e0327973. doi: 10.1371/journal.pone.0327973 (PMC12289039; doi:10.1371/journal.pone.0327973)

Figure S1: Validation of simulations with Orthoload

Hip and knee resultant joint contact force waveforms of squats (body weight only, barbell with 15kg, barbell with 35kg) of one participant were comparable to those obtained from a participant with an instrumented hip implant and another participant with an instrumented knee implant from the Orthoload database. The shape of the Orthoload waveforms were similar to the waveforms obtained from our athlete. It should be noted that the hip and knee joint contact force waveforms from the Orthoload database each were from one participant. Differences between our results and the values from Orthoload might be caused by a combination of differences in hip and knee kinematics, bone and muscle morphology, movement execution technique and velocities, additionally to the different methods to obtain the joint contact forces (simulations versus in-vivo measurement).


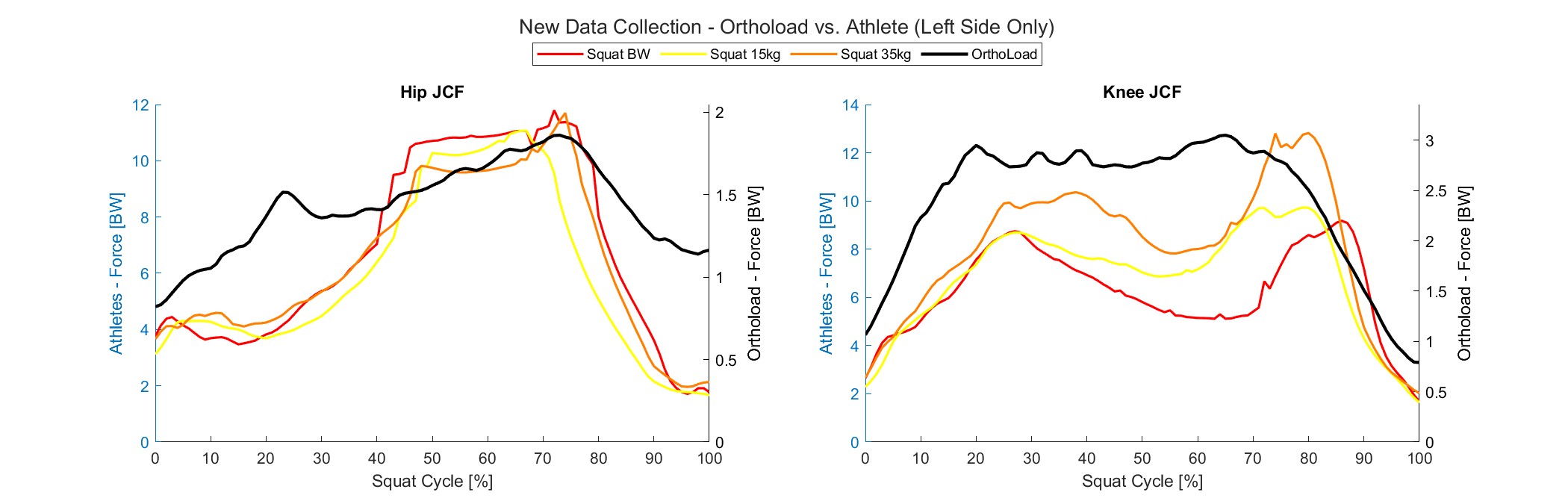

Supplement: S1 Fig — Hip and knee resultant joint contact force waveforms of squats (body weight only, 15 kg bar, 35 kg total) of one participant were comparable to those obtained from a participant with an instrumented hip implant and another participant with an instrumented knee implant from the Orthoload database. The shape of the Orthoload waveforms were similar to the waveforms obtained from our athlete. Visual comparison between the hip joint contact forces from the participants in our study with those found on the Orthoload database showed a reasonable agreement with our simulations. It should be noted that the hip and knee joint contact force waveforms from the Orthoload database each were from one participant. Differences between our results and the values from Orthoload might be caused by a combination of differences in hip and knee kinematics, bone and muscle morphology, movement execution technique and velocities, additionally to the different methods to obtain the joint contact forces (simulations versus in-vivo measurement). (DOCX) [file pone.0327973.s001.docx]
