# Supplementary material for: Biomechanical analysis of hip, knee, and ankle joint contact forces during squats in elite powerlifters
Source: PLoS One. 2025 Jul 24;20(7):e0327973. doi: 10.1371/journal.pone.0327973 (PMC12289039; doi:10.1371/journal.pone.0327973)

*Figure S2: Validation of simulations with Perrone et al. (2023).*

Hip resultant joint contact force waveforms of squats (body weight only) of one participant were comparable to single-legged squats obtained from the results of Perrone et al. (2023). The shape of this study’s waveforms was similar to the waveforms obtained from our athlete. The differences observed between our absolute values and the results reported by Perrone et al. (2023) may be attributable to several factors: firstly, the execution of single-legged half squats in the study by Perrone et al. (2023) instead of double-legged deep squats as in the present study. Secondly, the differences in the model employed, the movement execution technique, and the velocities may have contributed to the discrepancies in results.


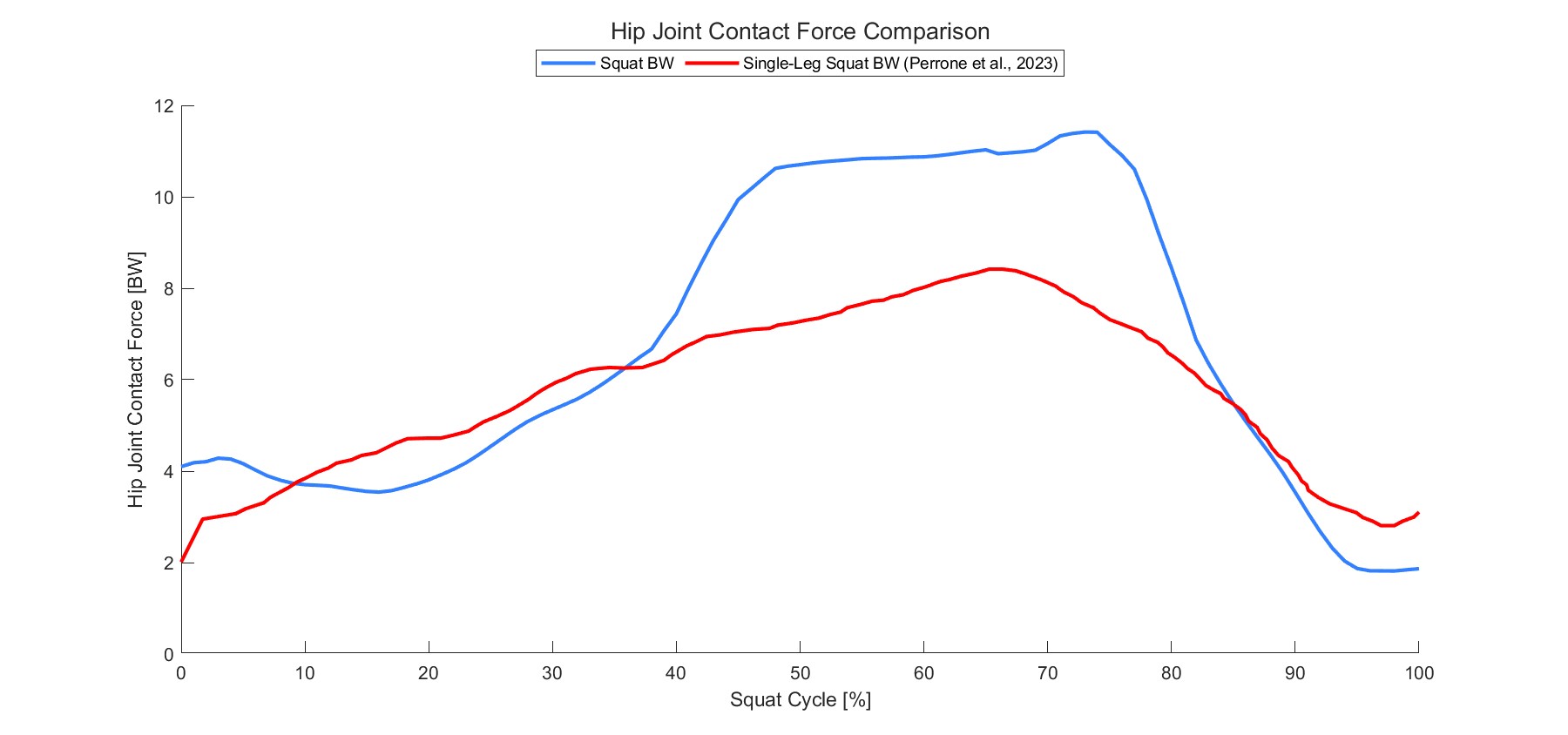

Supplement: S2 Fig — Hip resultant joint contact force waveforms of squats (body weight only) of one participant were comparable to single-legged squats obtained from the results of Perrone et al. (2023). The shape of this study’s waveforms was similar to the waveforms obtained from our athlete. The differences observed between our absolute values and the results reported by Perrone et al. (2023) may be attributable to several factors: firstly, the execution of single-legged half squats in the study by Perrone et al. (2023) instead of double-legged deep squats as in the present study. Secondly, the differences in the model employed, the movement execution technique, and the velocities may have contributed to the discrepancies in results. (DOCX) [file pone.0327973.s002.docx]
