# Supplementary material for: Biomechanical analysis of hip, knee, and ankle joint contact forces during squats in elite powerlifters
Source: PLoS One. 2025 Jul 24;20(7):e0327973. doi: 10.1371/journal.pone.0327973 (PMC12289039; doi:10.1371/journal.pone.0327973)

*Figure S3: Comparison of Muscle Activations Estimated via Static Optimization and Measured Using Surface Electromyography during the Squat Movement.*

0% and 100% of the squat cycle represent the upright standing position, while 50% corresponds to the lowest vertical position of the sacrum and thus the deepest point of the squat. Blue lines indicate muscle activations estimated via Static Optimization, and red lines represent muscle activations measured using surface electromyography (EMG). EMG signals were normalized to the peak activation value obtained from Static Optimization for comparability of waveform profiles.


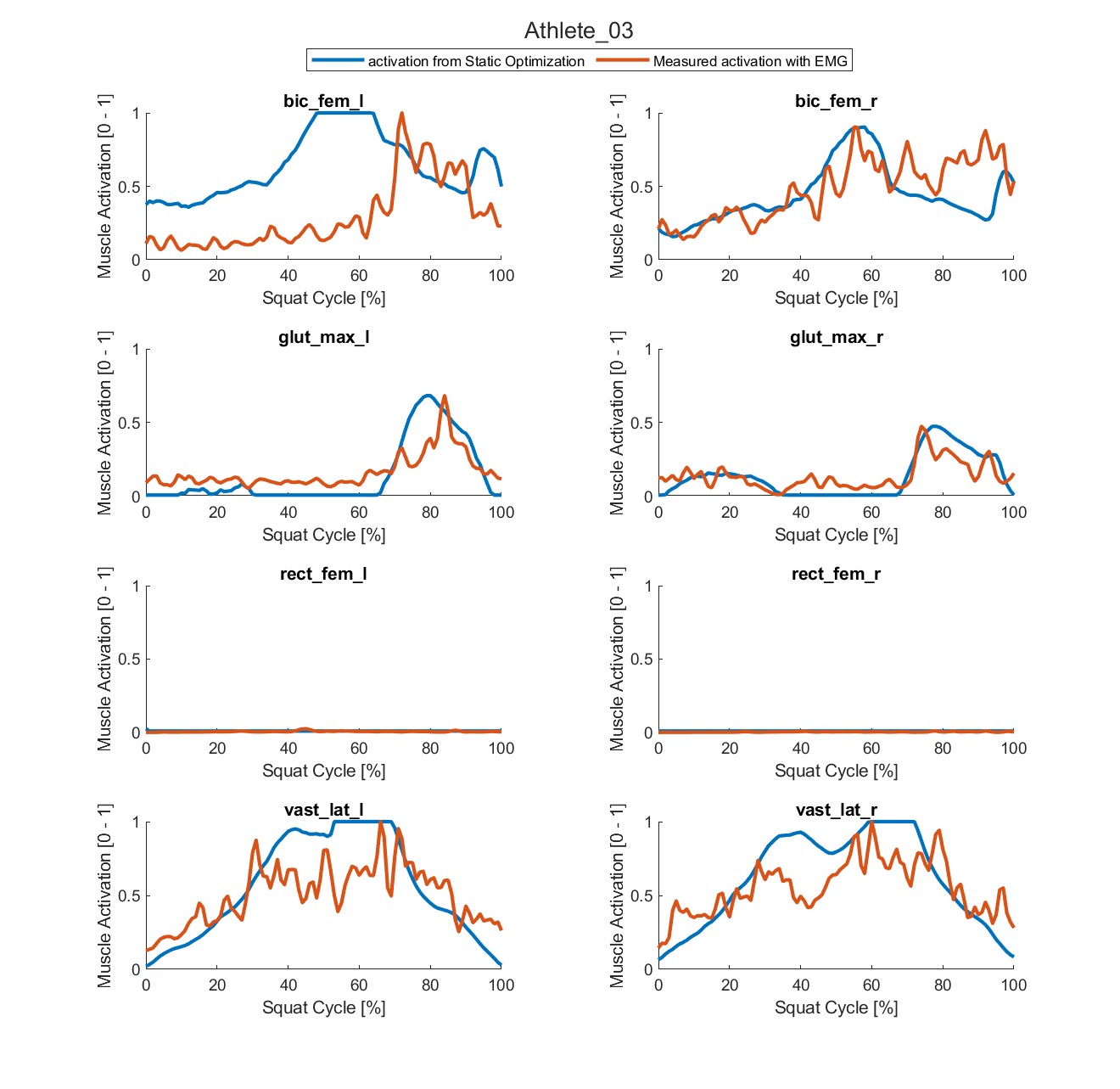

Supplement: S3 Fig — 0% and 100% of the squat cycle represent the upright standing position, while 50% corresponds to the lowest vertical position of the sacrum and thus the deepest point of the squat. Blue lines indicate muscle activations estimated via Static Optimization, and red lines represent muscle activations measured using surface electromyography (EMG). EMG signals were normalized to the peak activation value obtained from Static Optimization for comparability of waveform profiles. (DOCX) [file pone.0327973.s003.docx]
