# Supplementary material for: Biomechanical analysis of hip, knee, and ankle joint contact forces during squats in elite powerlifters
Source: PLoS One. 2025 Jul 24;20(7):e0327973. doi: 10.1371/journal.pone.0327973 (PMC12289039; doi:10.1371/journal.pone.0327973)
Supplement: S4 Fig — 0% and 100% of the squat cycle represent the respective upright position, and 50% denotes the lowest point of the sacrum, thus representing the deepest squat position. Solid lines in the figures represent the mean joint contact force across participants. Shaded areas represent ± one standard deviation. The lines below the figures indicate significant differences (SPM) during the squat cycle. The first four lines (light blue) represent significant differences between 70% 1-RM and 75% 1-RM, 70% 1-RM and 80% 1-RM, 70% 1-RM and 85% 1-RM, and 70% 1-RM and 90% 1-RM. The next three lines (orange) represent significant differences between 75% 1-RM and 80% 1-RM, 75% 1-RM and 85% 1-RM, and 75% 1-RM and 90% 1-RM. The following two lines (yellow) show significant difference between 80% 1-RM and 85% 1-RM, and 80% 1-RM and 90% 1-RM The last line (purple) shows significant differences between 85% 1-RM and 90% 1-RM. (DOCX) [file pone.0327973.s004.docx]

*Figure S4: Change of muscle forces throughout the squat cycle with different intensities.*

0% and 100% of the squat cycle represent the respective upright position, and 50% denotes the lowest point of the sacrum, thus representing the deepest squat position. Solid lines in the figures represent the mean joint contact force across participants. Shaded areas represent ± one standard deviation. The lines below the figures indicate significant differences (SPM) during the squat cycle. The first four lines (light blue) represent significant differences between 70 % 1-RM and 75 % 1-RM, 70 % 1-RM and 80 % 1-RM, 70 % 1-RM and 85 % 1-RM, and 70 % 1-RM and 90 % 1-RM. The next three lines (orange) represent significant differences between 75 % 1-RM and 80 % 1-RM, 75 % 1-RM and 85 % 1-RM, and 75 % 1-RM and 90 % 1-RM. The following two lines (yellow) show significant difference between 80 % 1-RM and 85 % 1-RM, and 80 % 1-RM and 90 % 1-RM The last line (purple) shows significant differences between 85 % 1-RM and 90 % 1-RM.


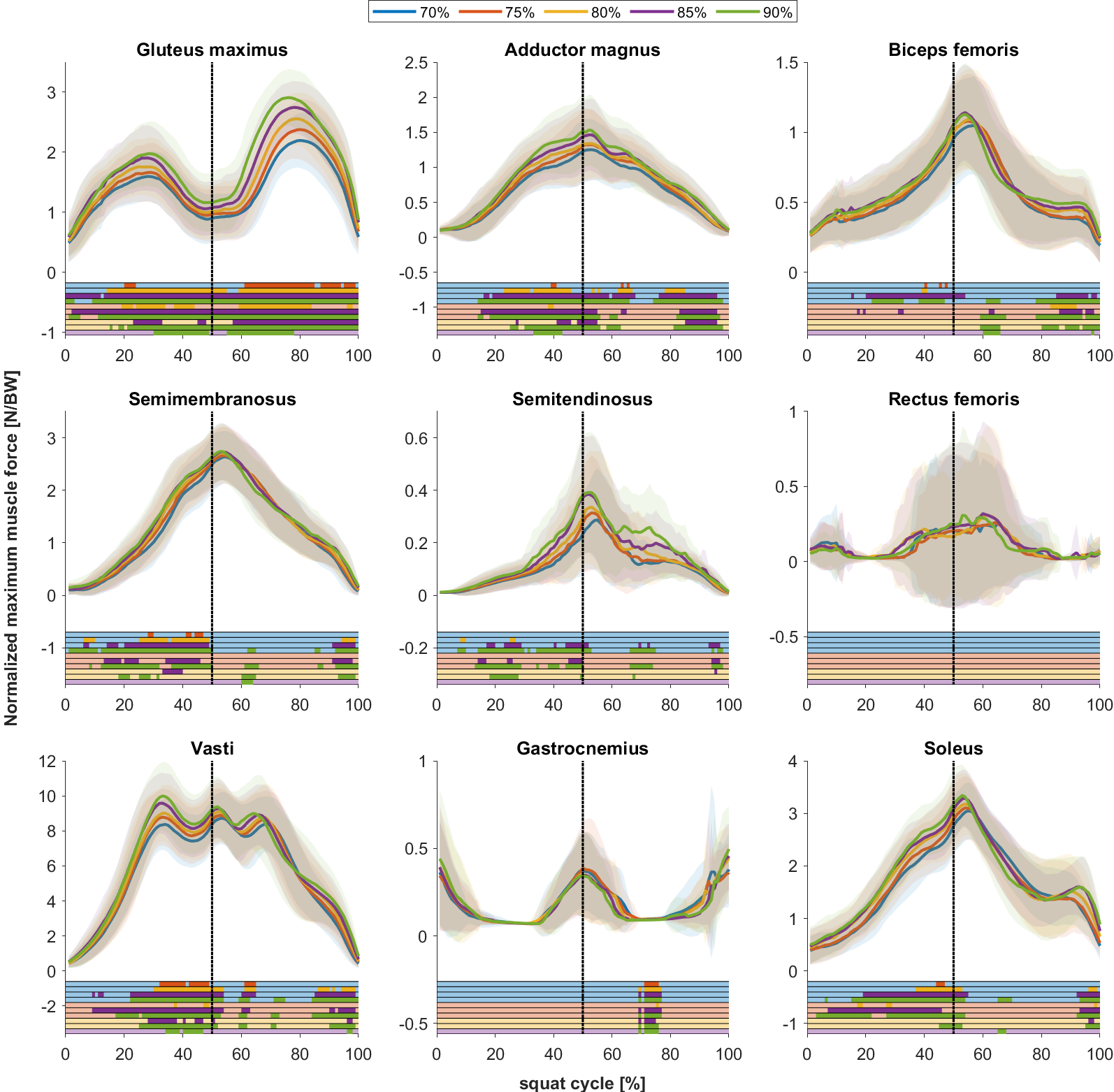


**[BW]**
