# Supplementary material for: Biomechanical analysis of hip, knee, and ankle joint contact forces during squats in elite powerlifters
Source: PLoS One. 2025 Jul 24;20(7):e0327973. doi: 10.1371/journal.pone.0327973 (PMC12289039; doi:10.1371/journal.pone.0327973)
Supplement: S5 Fig — a) Manual annotation of the target structures performed in the “Segment Editor” using the “Paint” function. At least ten slices per muscle were marked in each axis to provide a sufficient basis for the interpolation of the intermediate layers. b) “Fill between Slices” function was used to create a continuous segmentation. c) Where interpolation errors were noted, further slices were incorporated until a complete and anatomically precise segmentation was realised. (DOCX) [file pone.0327973.s005.docx]

*Figure S5: Muscle Segmentations using 3D slicer.*

1. Manual annotation of the target structures performed in the “Segment Editor” using the “Paint” function. At least ten slices per muscle were marked in each axis to provide a sufficient basis for the interpolation of the intermediate layers.
2. “Fill between Slices” function was used to create a continuous segmentation.
3. Where interpolation errors were noted, further slices were incorporated until a complete and anatomically precise segmentation was realized.


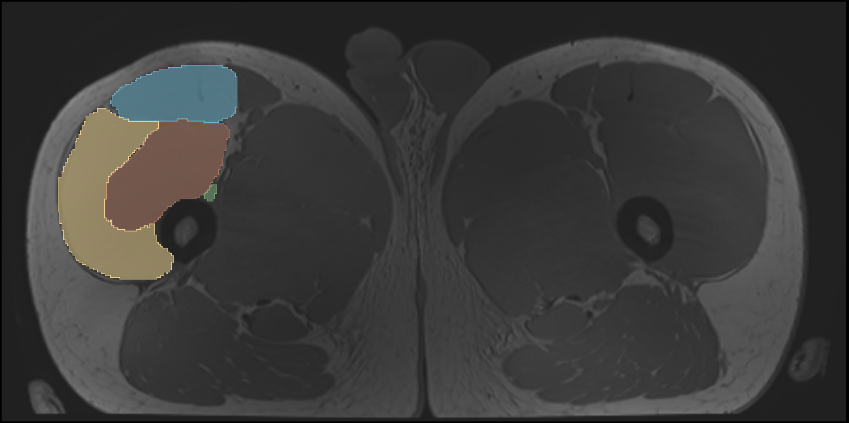


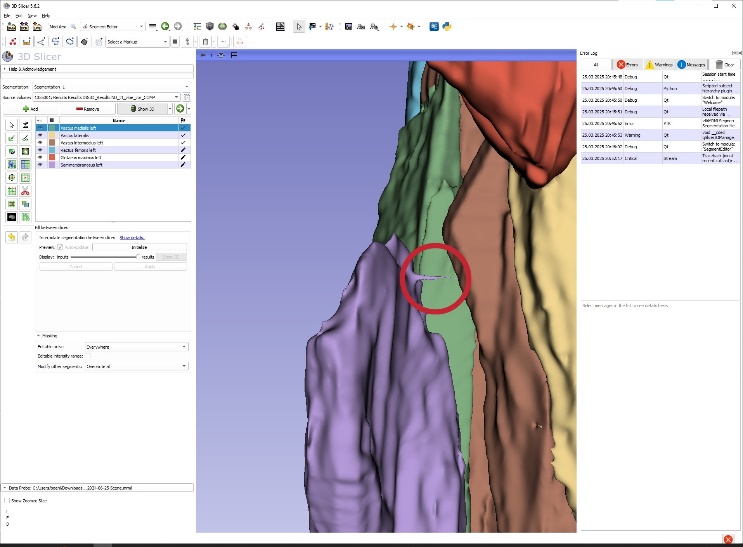


a


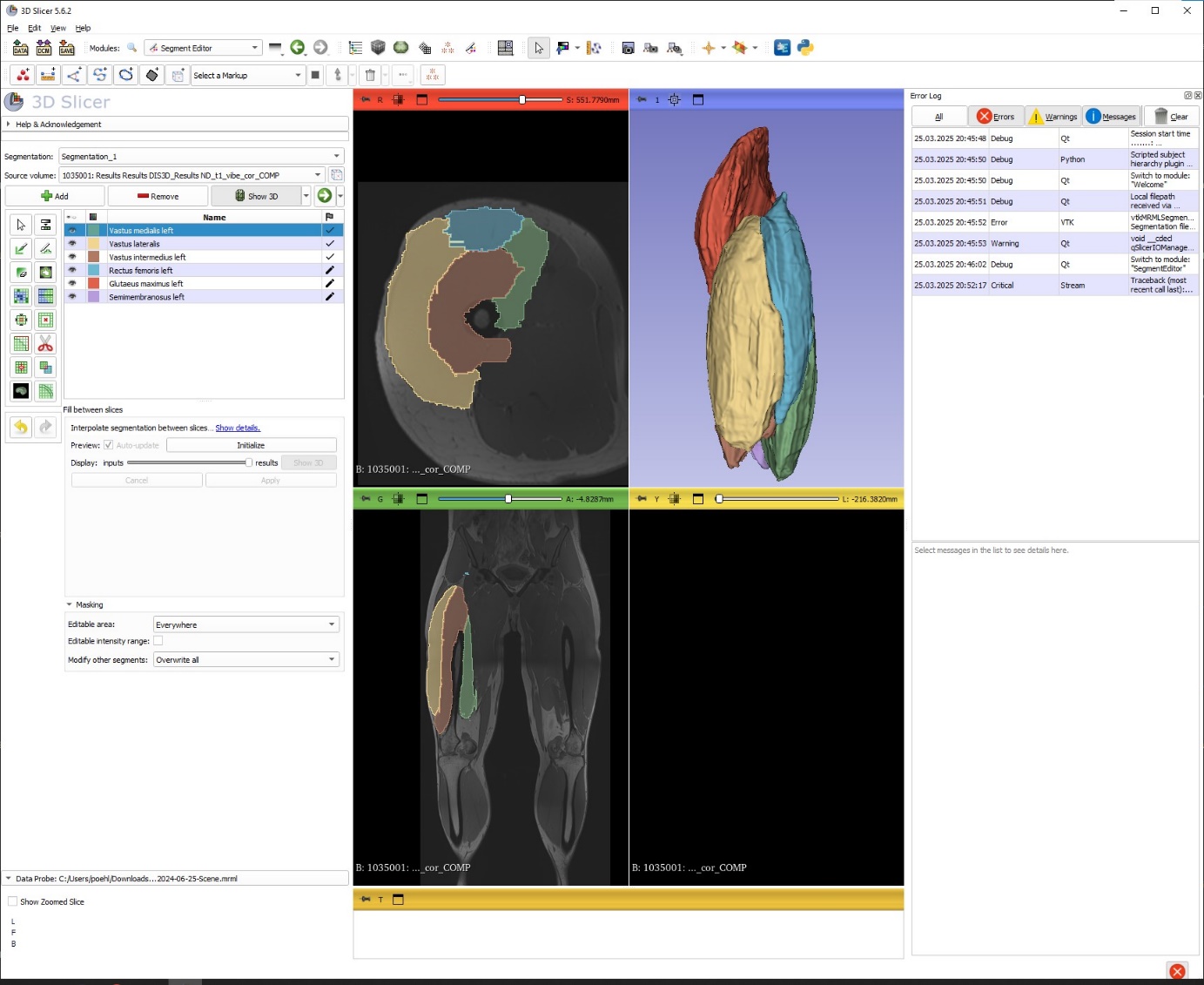

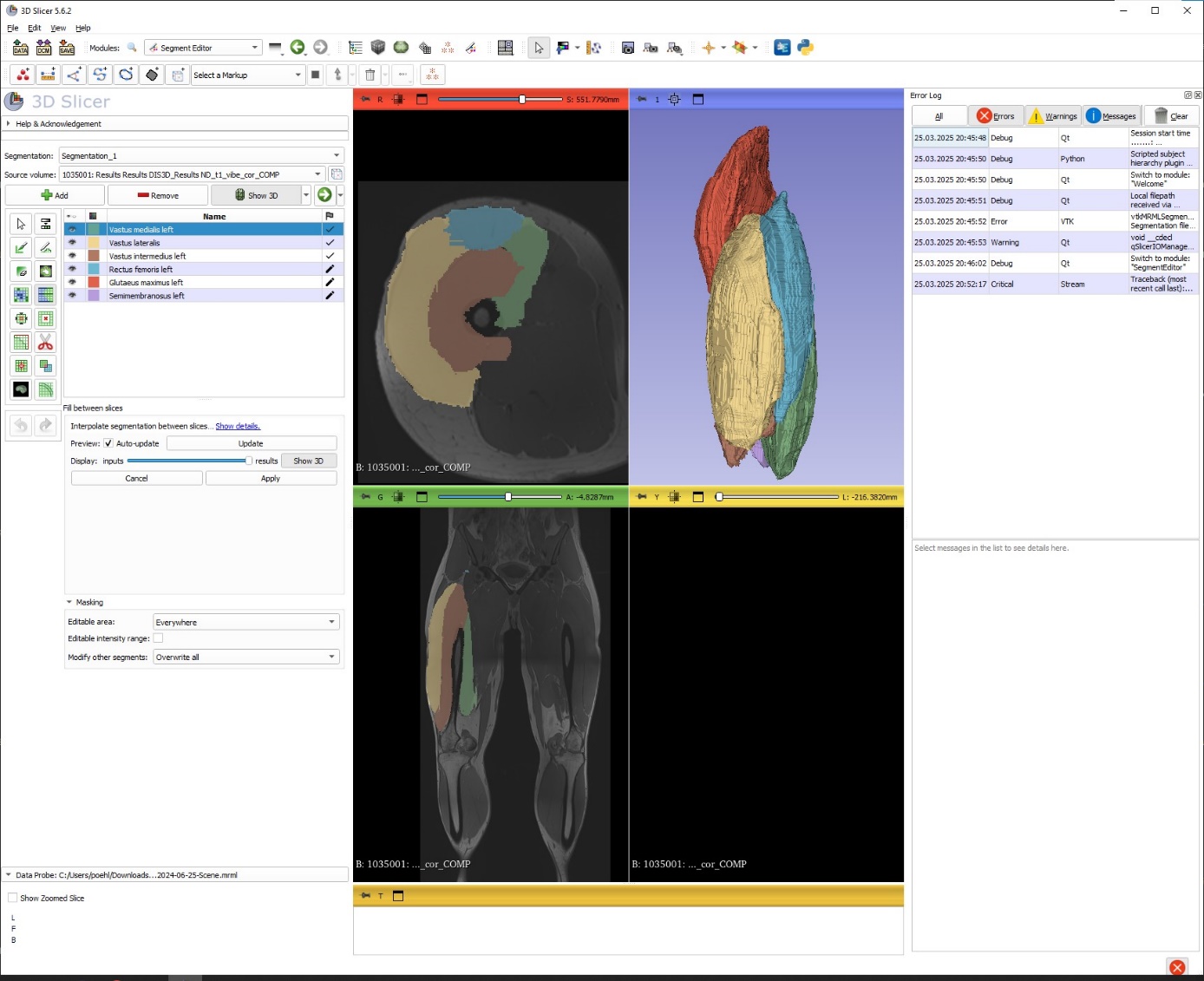

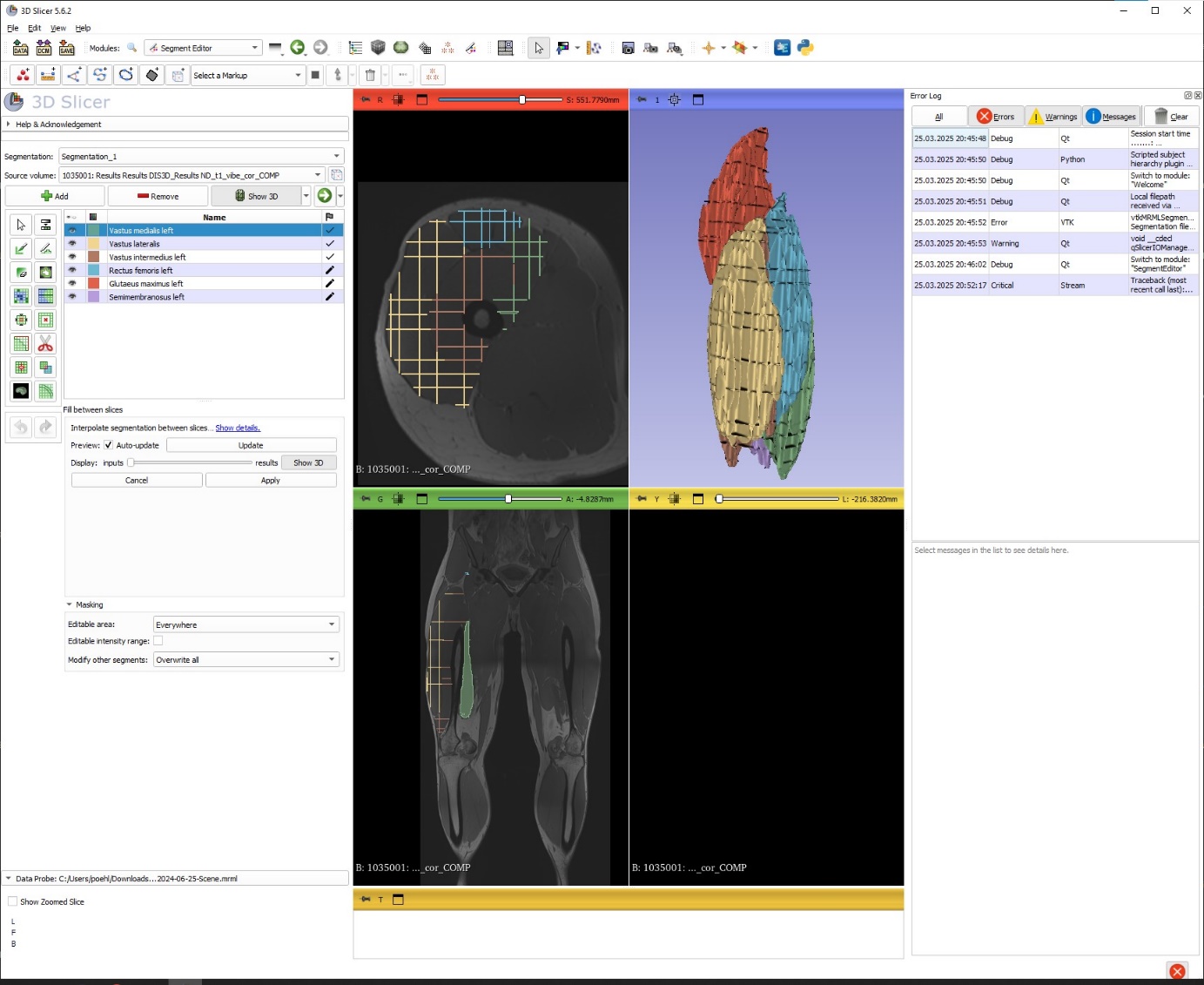


c

b
