# Supplementary material for: Biomechanical analysis of hip, knee, and ankle joint contact forces during squats in elite powerlifters
Source: PLoS One. 2025 Jul 24;20(7):e0327973. doi: 10.1371/journal.pone.0327973 (PMC12289039; doi:10.1371/journal.pone.0327973)
Supplement: S1 Table — 1-RM = 1-repetition maximum. (DOCX) [file pone.0327973.s006.docx]

Table S1: Percentage differences between the eccentric and concentric joint contact force during squats with increasing intensity (70%, 75%, 80%, 85%, 90% of 1-RM).

1-RM=1-repetition maximum

| Joint | Direction of force | 70% 1-RM | | 75% 1-RM | | 80% 1-RM | | 85% 1-RM | | 90% 1-RM | | Result for post-hoc test for phase | Result for post-hoc test for intensity |
| --- | --- | --- | --- | --- | --- | --- | --- | --- | --- | --- | --- | --- | --- |
|  |  | Ecc/Con | %Diff | Ecc/Con | %Diff | Ecc/Con | %Diff | Ecc/Con | %Diff | Ecc/Con | %Diff |  |  |
| Hip | ap | -1.55±0.34  -1.91±0.42 | **+23.2%±7.2** | -1.57±0.38  -1.95±0.40 | **+24.2%±7.7** | -1.63±0.44  -2.04±0.46 | **+25.2%±8.8** | -1.71±0.40  -2.13±0.45 | **+24.6%±7.7** | -1.76±0.42  -2.21±0.48 | **+25.6%±8.3** | Con > Ecc (absolute value)  (*p*<0.001*; η²_p_=0.513) | Increases in negative direction with intensity  (*p*<0.001*; η²_p_=0.113 |
|  | ml | 0.30±0.33  0.24±0.34 | **-20.0%±35.9** | 0.31±0.34  0.25±0.34 | **-19.4%±33.8** | 0.27±0.28  0.26±0.26 | **-3.7%±5.3** | 0.32±0.39  0.34±0.39 | **+6.3%±10.5** | 0.34±0.37  0.36±0.41 | **+5.9%±9.3** | Con = Ecc  (p=0.632; η²_p_=0.003) | Increases with intensity  (p=0.004*; η²_p_=0.059) |
|  | v | 12.95±3.20  13.63±3.07 | **+5.3%±1.8** | 13.37±3.28  13.98±3.06 | **+4.6%±1.5** | 13.63±3.03  14.33±2.98 | **+5.1%±1.6** | 14.33±2.98  15.05±3.05 | **+5.0%±1.5** | 14.65±3.05  15.36±2.97 | **+4.8%±1.4** | Con > Ecc  (*p*<0.001*; η²_p_=0.122) | Increases with intensity  (*p*<0.001*; η²_p_=0.450) |
|  | r | 13.03±3.21  13.70±3.08 | **+5.1%±1.7** | 13.45±3.29  14.06±3.07 | **+4.5%±1.5** | 13.77±3.03  14.42±2.98 | **+4.7%±1.4** | 14.46±3.12  15.16±3.05 | **+4.8%±1.4** | 14.73±3.05  15.46±2.97 | **+5.0%±1.4** | Con > Ecc  (*p*<0.001*; η²_p_=0.123) | Increases with intensity  (*p*<0.001*; η²_p_=0.449) |
| Tibio-femoral | ap | 12.17±2.38  12.79±2.35 | **+5.1%±1.4** | 12.59±2.35  13.02±2.27 | **+3.4%±0.9** | 12.91±2.46  13.35±2.41 | **+3.4%±0.9** | 13.31±2.42  13.69±2.32 | **+2.9%±0.7** | 13.50±2.50  13.82±2.44 | **+2.4%±0.6** | Con > Ecc  (p<0.001*; η²_p_=0.129) | Increases with intensity  (p<0.001*; η²_p_=0.512) |
|  | ml | 0.03±0.09  0.08±0.08 | **+167%±89** | 0.04±0.10  0.08±0.10 | **+100%±53** | 0.04±0.09  0.10±0.13 | **+150%±80** | 0.04±0.08  0.10±0.11 | **+150%±77** | 0.05±0.11  0.12±0.13 | **+140%±67** | Con > Ecc  (p=0.005*; η²_p_=0.148) | Stays constant with intensity  (p=0.079; η²_p_=0.017) |
|  | v | 17.27±2.77  17.72±3.36 | **+2.6%±1.0** | 18.31±2.97  18.23±3.33 | **-0.4%±0.9** | 18.89±2.99  18.84±3.84 | **-0.3%±0.9** | 19.90±3.40  19.28±3.94 | **-3.1%±1.0** | 20.51±3.49  19.85±3.94 | **-3.2%±1.0** | Con = Ecc  (p=0.396; η²_p_=0.005) | Increases with intensity  (p<0.001*; η²_p_=0.480) |
|  | r | 19.50±3.10  20.74±3.75 | **+6.4%±1.6** | 20.75±3.34  21.41±3.69 | **+3.2%±0.9** | 21.46±3.33  22.13±4.14 | **+3.1%±0.8** | 22.49±3.68  22.59±4.13 | **+2.0%±0.6** | 23.22±3.86  23.13±4.35 | **-1.2%±0.4** | Con > Ecc  (*p*=0.021*; η²_p_=0.031) | Increases with intensity  (p<0.001*; η²_p_=0.531) |
| Patello-femoral | ap | 18.92±3.03  19.77±3.51 | **+4.5%±1.8** | 20.11±3.24  20.45±3.51 | **+1.7%±0.7** | 20.72±3.26  21.07±3.90 | **+1.7%±0.7** | 21.74±3.65  21.51±3.90 | **-1.1%±0.5** | 22.40±3.80  21.90±4.15 | **-2.2%±1.1** | Con = Ecc  (*p*=0.396; η²_p_=0.004) | Increases with intensity  (p<0.001*; η²_p_=0.533) |
|  | ml | 0.24±0.21  0.21±0.22 | **-12.5%±15.3** | 0.26±0.22  0.24±0.19 | **-7.7%±9.2** | 0.28±0.24  0.25±0.22 | **-10.7%±10.3** | 0.30±0.24  0.26±0.21 | **-13.3%±11.7** | 0.29±0.23  0.28±0.23 | **-3.4%±2.9** | Con = Ecc  (p=0.092; η²_p_=0.016) | Stays constant with intensity  (p=0.07; η²_p_=0.051) |
|  | v | 13.15±2.16  13.60±2.14 | **+3.4%±1.2** | 13.86±2.24  14.00±2.29 | **+1.0%±0.4** | 14.14±2.11  14.26±2.28 | **+0.8%±0.3** | 14.71±2.16  14.57±2.22 | **-0.9%±0.4** | 15.02±2.32  14.82±2.31 | **-1.3%±0.5** | Con = Ecc  (p=0.342; η²_p_=0.003) | Increases with intensity  (p<0.001*; η²_p_=0.591) |
|  | r | 22.80±3.57  23.83±3.95 | **+4.5%±1.2** | 24.17±3.82  24.61±4.00 | **+1.8%±0.5** | 24.88±3.75  25.27±4.35 | **+1.6%±0.5** | 26.07±4.11  25.77±4.31 | **-1.1%±0.4** | 26.73±4.30  26.25±4.64 | **-1.8%±0.5** | Con = Ecc  (*p*=0.279; η²_p_=0.005) | Increases with intensity  (p<0.001*; η²_p_=0.559) |
| Ankle | ap | -4.55±1.31  -4.90±1.32 | **+7.7%±2.1** | -4.73±1.47  -5.04±1.43 | **+6.6%±2.1** | -4.87±1.42  -5.12±1.36 | **+5.2%±1.6** | -5.03±1.37  -5.29±1.33 | **+5.1%±1.5** | -5.19±1.55  -5.44±1.48 | **+4.8%±1.6** | Con > Ecc (absolute value)  (p<0.001*; η²_p_=0.128) | Increases in negative direction with intensity  (*p*=0.001; η²_p_=0.270) |
|  | ml | 0.25±0.14  0.31±0.15 | **+24.0%±10.3** | 0.25±0.14  0.28±0.14 | **+12.0%±5.2** | 0.26±0.15  0.29±0.16 | **+11.5%±5.3** | 0.28±0.14  0.32±0.15 | **+14.3%±6.0** | 0.30±0.16  0.35±0.17 | **+16.7%±6.5** | Con > Ecc  (p=0.002*; η²_p_<0.104) | Increases with intensity  (p<0.001*; η²_p_=0.089) |
|  | v | 8.41±1.56  9.20±1.71 | **+9.4%±2.3** | 8.82±1.92  9.33±1.78 | **+5.8%±1.4** | 9.03±1.78  9.58±1.72 | **+6.1%±1.5** | 9.26±1.67  9.77±1.48 | **+5.5%±1.3** | 9.57±2.01  10.14±1.92 | **+6.0%±1.4** | Con > Ecc  (p<0.001*; η²_p_=0.135) | Increases with intensity  (p<0.001*; η²_p_=0.204) |
|  | r | 9.59±1.93  10.44±1.92 | **+8.9%±2.3** | 10.03±2.33  10.61±2.13 | **+5.8%±1.5** | 10.29±2.16  10.82±2.00 | **+5.1%±1.4** | 10.57±2.03  11.13±1.76 | **+5.3%±1.3** | 10.91±2.43  11.48±2.21 | **+5.2%±1.2** | Con > Ecc  (p<0.001*; η²_p_=0.137) | Increases with intensity  (p<0.001*; η²_p_=0.239) |
