# Supplementary material for: Biomechanical analysis of hip, knee, and ankle joint contact forces during squats in elite powerlifters
Source: PLoS One. 2025 Jul 24;20(7):e0327973. doi: 10.1371/journal.pone.0327973 (PMC12289039; doi:10.1371/journal.pone.0327973)
Supplement: S2 Table — 1-RM = 1-repetition maximum. (DOCX) [file pone.0327973.s007.docx]

Table S2: 1-RM values and gender of the participants.

1-RM=1-repetition maximum.

| **Participant** | **Gender** | **1-RM Squat (kg)** | **1-RM Squat (xBM)** |
| --- | --- | --- | --- |
| **Athlete 1** | w | 120 | 2.1 |
| **Athlete 2** | w | 122.5 | 1.9 |
| **Athlete 3** | m | 250 | 2.9 |
| **Athlete 4** | w | 135 | 1.7 |
| **Athlete 5** | m | 285 | 2.9 |
| **Athlete 6** | w | 132.5 | 1.7 |
| **Athlete 7** | w | 130 | 1.9 |
| **Athlete 8** | w | 125 | 2.1 |
| **Athlete 9** | w | 135 | 2.1 |
| **Athlete 10** | m | 250 | 3.0 |
| **Athlete 11** | m | 237.5 | 2.6 |
| **Athlete 12** | m | 280 | 3.0 |
| **Athlete 13** | m | 250 | 2.9 |
| **Athlete 14** | m | 240 | 2.5 |
| **Athlete 15** | m | 232.5 | 2.7 |
| **Athlete 16** | w | 140 | 2.3 |
| **Athlete 17** | m | 242.5 | 2.6 |
| **Athlete 18** | w | 120 | 2.2 |
| **Athlete 19** | m | 300 | 2.7 |
| **Athlete 20** | m | 280 | 2.9 |
| **Athlete 21** | w | 155 | 2.1 |
| **Athlete 22** | w | 122.5 | 1.9 |
| **Athlete 23** | m | 257.5 | 3.0 |
| **Athlete 24** | m | 277.5 | 2.8 |
| **Athlete 25** | m | 240 | 2.0 |
| **Athlete 26** | w | 102.5 | 1.8 |
| **Athlete 27** | w | 125 | 2.4 |
| **Athlete 28** | m | 222.5 | 2.2 |
| **Athlete 29** | m | 255 | 2.1 |
