# Supplementary material for: Biomechanical analysis of hip, knee, and ankle joint contact forces during squats in elite powerlifters
Source: PLoS One. 2025 Jul 24;20(7):e0327973. doi: 10.1371/journal.pone.0327973 (PMC12289039; doi:10.1371/journal.pone.0327973)
Supplement: Attachment — Submitted filename: Response_to_Reviewer_JCF_Powerlifter.docx [file pone.0327973.s008.docx]

**Dear Editor,**

**Dear Reviewers,**

Please allow us to express our sincere gratitude for the considerable time you have dedicated to the initial review, which was tremendously beneficial in enhancing the manuscript. It is our understanding that the constructive and valuable input that has been provided has resulted in a significant enhancement in the quality of the work.

We would also like to express our gratitude for the new feedback, which has helped to make the manuscript even more scientifically rigorous. We have done our utmost to address every single point.

We are pleased to say that, after careful consideration, we are now confident that the manuscript has addressed all the concerns raised and meets the high standards required for publication in PLoS One.

Thank you again for your guidance and support. We look forward to your feedback on our resubmission.

Warm regards,

Alexander Pürzel

Corresponding author

**Comments and Responses:**

**Reviewer 1:**

| **Nr.** | **Comment** | **Response** |
| --- | --- | --- |
| 0 | Thank you for your detailed and thoughtful responses to the comments. The revisions have significantly enhanced the manuscript’s clarity, scientific rigor, and practical relevance.  I especially appreciate the expanded discussion on cartilage adaptation, the improved methodological transparency, and the strengthened implications for training and injury prevention. Including EMG-based cross-validation and providing a clear justification for using the validated Catelli model without modifications demonstrate strong scientific integrity. The additional details on MRI volume calculation and the explanation of the force-length-velocity relationship further enrich the manuscript.  Your efforts to make the findings more accessible and applicable to both research and practice are commendable.  Well done. | We would like to sincerely thank you for your time, effort, and expertise in reviewing our manuscript. Your thoughtful and detailed comments were highly valuable in guiding our revisions. We are grateful for your positive review and pleased that the changes you suggested improved the clarity, scientific quality and practical relevance of the manuscript. Your feedback was both encouraging and motivating.  Thank you again for your support. |

**Reviewer 2:**

Changes in the manuscript following reviewer 2’s comments are written in red.

| **Nr.** | **Comment** | **Response** |
| --- | --- | --- |
| 0 | The authors substantially revised this manuscript to address the prior concerns, and I command on their diligent efforts. My primary concern on the original paper was the validity of the models and their high-magnitude JCF estimates. In this revision, the authors added new validation data from one participant during bodyweight squats, compared the JCF estimates to a recent study on this same exercise, and included EMG data from one participant to compare with the model estimates. Even with n = 1, these data still increased my confidence in the models. The authors also emphasized in Discussion the limitations on validation and caution to the result data, which are important and necessary additions. With these, I have been convinced that the authors now have the minimum necessary evidence for the validity of their models to be deemed acceptable. Beyond model validation, the authors also greatly improved the clarity of their paper, and most of my previous concerns on clarity have been resolved. I also agree that the authors' additional discussions in response to the other reviewer will enhance the value of this work. Overall, I am satisfied with the quality of the revised work, and I have no other major concerns. Several minor points of ambiguity or confusion remains that I think should be corrected or explained – please see below. Page [P] and line numbers [L] refer to the revised manuscript with tracked changes. | We are very grateful for your thoughtful and constructive feedback, as well as the time and expertise you dedicated to reviewing our revised manuscript. Your recognition of our efforts to improve model validation, address key concerns, and enhance the clarity of the paper is greatly appreciated. We are especially encouraged by your assessment that the revisions have sufficiently addressed your main concerns regarding model validity.  Regarding the remaining minor points of ambiguity or confusion, we carefully reviewed each of them and did our best to clarify and improve these aspects in the manuscript. Thank you again for your valuable input and support throughout the review process. |

| **Nr.** | **Comment** | **Response** |
| --- | --- | --- |
| 1 | [P4/L58]: For clarity, replace “the movement” with “anterior translation”. | We agree. That is a much more appropriate and understandable expression.  Line 57: Furthermore, compressive forces are of paramount importance for stabilising the knee joint, as they counteract anterior-posterior shear forces and, therefore, reduce the anterior translation of the tibia in relation to the femur while also contributing to frontal plane stability by limiting varus and valgus motion [23]. |
| 2 | [P6/L108]: 73 and 55 do not subtract down to 20 calibration-only markers. | Thank you for pointing out this typo. We have corrected it.  Line 107: A three-dimensional motion capture system comprising 12 cameras (Vicon Motion System, Oxford, UK) was used to capture the trajectories of 73 (static position) and 53 (squat movement) markers on the lower limbs, upper body, and barbell with a recording frequency of 200 Hz (Figure 1). |
| 3 | [P9/L181]: While the new descriptions clarified that Equation 2 was used to determine the scale factor S, it is confusing that this is not represented in the equations, leaving Equation 1 and 2 disjointed. Would it be correct to formulate that S ~= mean (F_iso / F_scaled_equation_1) = 3 (with n = 6), then F_scaled_final = F_scaled_equation_1 * S (for n = 29)? Also, please add back the caption that “ss” means subject specific. | Thank you for the advice on making this section clearer and easier to understand. We have taken on board your valuable feedback for the manuscript and revised the section accordingly.  Line 162: In order to ensure that the model is capable of producing necessary internal joint moments to resist the external moments, the maximum isometric muscle forces ($F$) for all models were adjusted based on their body mass ($M$) using Equation 1 [30, 31].  Equation 1 $F_{iso,scaled}=F_{iso,generic}*\left( \frac{M_{scaled}}{M_{generic}} \right)^{\frac{2}{3}}$  Despite this muscle force scaling process, the simulations resulted in reserve actuator forces that were beyond 10 % of the net joint moments, thereby producing simulations that were not realistic [32]. In other words, the models were too weak to perform the high load exercises of the elite-level athletes. In order to obtain a more precise estimation of the isometric maximum force of the participants and to make the necessary adjustments to the models, MRI scans of the lower limbs were analysed from six participants. The vasti, rectus femoris, semimembranosus, and gluteus maximus muscles were segmented and their volumes were calculated using the 3D Slicer 5.2.2 [26]. The maximum isometric muscle force was subsequently calculated using Equation 2 [33, 34], where $F_{iso}$ represents the muscle's isometric maximum force, $ss$ means subject specific, $V$ is the muscle volume determined by the MRI scans and $l_{o}^{m}$ is the optimal fibre length (OpenSim 4.4 default [27]). The maximum fibre tension (σ) was set to 105 N cm⁻², a value which falls within the range previously reported in the literature [35]. This choice was based on anatomical cross-sectional areas measured in bodybuilders [36] and was adjusted for muscle pennation angle [36, 37, 38] to better reflect the physiological cross-sectional area.  Equation 2 $F_{iso, ss}=\frac{V_{ss}}{l_{o,ss}^{m}}*\sigma$  The maximum isometric force calculated using Equation 1 was found to be three times lower ($S$ obtained with Equation 3) than the corresponding estimate using Equation 2, which was based on MRI-derived muscle volume data.  Equation 3 $S\approx mean \frac{F_{iso,ss}}{F_{iso,scaled}}$  Consequently, the final equation (Equation 4) employed for the scaling of isometric forces across all 29 models involved multiplying the initial equation (Equation 1) by factor $S$ (Equation 3), to ensure that the models are capable of withstanding the external loads.  Equation 4 $F_{iso,scaled\_final}=F_{iso,generic}*\left( \frac{M_{scaled}}{M_{generic}} \right)^{\frac{2}{3}}*S$  Although Equation 2 was applied only to the muscle volume values of the MRI subgroup (6 athletes), its output was used to define the scaling factor in Equation 4. Given the homogeneity of the participants with respect to their performance level, Equation 4 with $S=3$ was employed to scale the isometric maximum force for the models of all 29 participants. |
| 4 | [P10/L208]: Were EMG data also acquired from the unloaded squat, or from a sub-1-RM loaded squat? Were the EMG and JCF validation data from the same participant? Please clarify. | Thank you for this notice. We have clarified the statement.  Line 212: Furthermore, for the same participant, electromyography (EMG) data were available and used as an additional validation step for evaluating the plausibility of the simulated neuromuscular control patterns during squatting. For the 70 % 1-RM condition, we qualitatively compared EMG signals of m. biceps femoris, m. gluteus maximus, m. rectus femoris, and m. vastus lateralis with muscle activation output from our musculoskeletal simulations (i.e., obtained from Static Optimization). |
| 5 | [P18/L294]: In the updated Figure 3, it appears that hip JCF is now medial (-), not lateral. | Thank you. We very much appreciate it, and have changed the wording in the manuscript to “medially”.  Line 300: The medio-lateral forces, which showed only minimal changes with increasing intensities, were small compared to the other force components and had a medially oriented vector. |
| 6 | [P21/L358]: Why is the patellofemoral JCF described again here, with a different value from the previous sentence? Please double check, as citation [49] seems to be a study on hip JCF. | Thank you for pointing out this typo. We have corrected it.  Line 364: In the hip joint, forces of approximately 2 BW were observed during squats without additional loads [49]. |
| 7 | [Table 1]: For clarity and consistency, can you simply replace “relative squat performance” with “one-repetition maximum (1-RM)”? | We agree and have replaced the term as suggested by the reviewer. |
| 8 | [Figures 2 and 3]: I was able to access the original TIFF figures, and they are of good resolution. What do the shapes of the shaded areas represent? Are these violin plots? Also clarify what the shaded areas around the Figure 3 waveforms represent. | Thank you for pointing out the missing information. The shaded areas are violin plots representing the distribution of data for each condition using kernel density estimation. The darker shaded sections indicate the interquartile range, while the lighter sections represent the remainder of the distribution. We have updated the figure caption to clarify this.  Line 261: Shaded areas represent violin plots, illustrating the distribution of data; darker areas indicate quartiles, while lighter areas show the remainder of the distribution.  In Figure 3, the shaded areas around each waveform represent ±1 standard deviation. We have added this explanation to the figure caption for clarity.  Line 285: Solid lines in the figures represent the mean joint contact force across participants. Shaded areas represent ± one standard deviation. |
| 9 | [Figure S1]: Use black color on the right-side Y scale to match the correct data line. Change the yellow line label to “Squat 15kg”. | We agree. That makes the figure much easier to understand. Thank you very much. We have adapted Figure S1 accordingly. |
| 10 | [Figure S2]: Does each of these represent double- or single-legged squats? I would expect JCFs to approximately double when countering full versus half body weight. | Thank you very much for this input. The study by Perrone et al. (2023) involved single-legged squats, our study involved double-legged squats. We have now updated the label on Figure S2 to reflect this. The fact that the participants in the Perrone et al. (2023) study performed only half squats that were not as deep as in our study may explain why the absolute values are lower despite the one-legged performance. Therefore, we have revised the caption to clarify that the Perrone et al. (2023) study involved single-legged half squats, which could explain the lower absolute hip joint contact forces observed.  Caption Figure S2: Hip resultant joint contact force waveforms of squats (body weight only) of one participant were comparable to single-legged squats obtained from the results of Perrone et al. (2023). The shape of this study’s waveforms was similar to the waveforms obtained from our athlete. The differences observed between our absolute values and the results reported by Perrone et al. (2023) may be attributable to several factors: firstly, the execution of single-legged half squats in the study by Perrone et al. (2023) instead of double-legged deep squats as in the present study. Secondly, the differences in the model employed, the movement execution technique, and the velocities may have contributed to the discrepancies in results. |
| 11 | [Figure S3]: Please set all Y scales to 0–1. Model-estimated rectus femoris activation appears on this figure as if a large asymmetry existed, but the finding was that they were both near-zero. | We agree. We have adapted Figure S3 accordingly. |
| 12 | [Figure S4]: Update figure caption to clarify what the waveform shadings and SPM lines mean. | Thank you for the comment. We updated the figure caption.  Figure S4: 0% and 100% of the squat cycle represent the respective upright position, and 50% denotes the lowest point of the sacrum, thus representing the deepest squat position. Solid lines in the figures represent the mean joint contact force across participants. Shaded areas represent ± one standard deviation. The lines below the figures indicate significant differences (SPM) during the squat cycle. The first four lines (light blue) represent significant differences between 70 % 1-RM and 75 % 1-RM, 70 % 1-RM and 80 % 1-RM, 70 % 1-RM and 85 % 1-RM, and 70 % 1-RM and 90 % 1-RM. The next three lines (orange) represent significant differences between 75 % 1-RM and 80 % 1-RM, 75 % 1-RM and 85 % 1-RM, and 75 % 1-RM and 90 % 1-RM. The following two lines (yellow) show significant difference between 80 % 1-RM and 85 % 1-RM, and 80 % 1-RM and 90 % 1-RM The last line (purple) shows significant differences between 85 % 1-RM and 90 % 1-RM. |
| 13 | [Table S2]: It may be useful to also report these values as normalized (xBM) in addition to kg. | We agree and also reported normalized (xBM) values in addition to kg in the Table S2 of the revised manuscript. |
